# Deletion of *dltD* gene modulates biofilm matrix and acid metabolism to attenuate *Streptococcus mutans* cariogenicity

**DOI:** 10.3389/fcimb.2025.1741359

**Published:** 2026-02-16

**Authors:** Jingyun Du, Shan Huang, Yijun Li, Zhoucheng Qiu, Yujia Hao, Jing Huang, Ling Zhan, Shuai Chen, Xiaojing Huang

**Affiliations:** 1 Clinical Research Center for Oral Tissue Deficiency Diseases of Fujian Province & Fujian Key Laboratory of Oral Diseases & Fujian Provincial Engineering Research Center of Oral Biomaterial, School and Hospital of Stomatology, Fujian Medical University, Fuzhou, China; 2 Stomatological Key Laboratory of Fujian College and University & Institute of Stomatology & Research Center of Dental and Craniofacial Implants, School and Hospital of Stomatology, Fujian Medical University, Fuzhou, China; 3 Stomatology College, Quanzhou Medical College, Quanzhou, China; 4 Division of Pediatric Dentistry, Department of Orofacial Sciences, Department of Preventive and Restorative Dental Sciences, University of California, San Francisco, San Francisco, CA, United States

**Keywords:** acid production, acid tolerance, biofilm, *dltD*, *Streptococcus mutans*

## Abstract

**Introduction:**

*Streptococcus mutans (SM)* is one of the key pathogenic bacteria in the occurrence and development of dental caries. Its complex virulence regulation network has become an important target in current ecological caries prevention research. This study explored how *dltD* attenuates *SM* cariogenicity using standard strain *SM*UA159, high-cariogenic clinical strain *SM*593, and their *dltD* deletion/complemented strains.

**Methods:**

In this study, the clinical serotype C *SM*593 clinical strain isolated from caries-active patients (DMFT6), the *SM*593 dltD deletion strain (*SM*593-*dltD*), and *SM*593-*dltD* complementary strain (*SM*593-*dltD-c*) were selected as the experimental strains. Rat caries model was constructed to detect the cariogenicity. Colony forming counting units (CFU) counting was used to detect the colonization ability *in vivo*. The adhesion ability and surface hydrophobicity of each strain were examined by tube attachment assay and microbial adhesion to hydrocarbons method. Biofilm of each strain was constructed *in vitro*., CFU counting and MTT staining were used to analyze the *SM* biofilm formation. Laser confocal scanning microscope were used to observe the biofilm morphology, live/dead staining distribution. Anthrone-sulfuric acid assay, laser confocal scanning microscope, SYTOX probe assay and BCA protein kit assay were used to detect the extracellular polysaccharide content, extracellular polysaccharide distribution, eDNA content and extracellular protein content of the biofilm. Acid production was examined by detecting the pH of the biofilm supernatant. Potassium iodide assay and lactate dehydrogenase detection kit assay were used to examine intracellular polysaccharides and lactate dehydrogenase activity. CFU counting was used to detect the adaptive acid tolerance ability. Laurdan fluorescent probe was used to examine the cell membranes fluidity under the acidic condition. The expression of genes related to biofilm formation and acid tolerance was detected by RTqPCR.

**Results:**

*In vivo*, *dltD* deletion significantly reduced fissure and proximal caries severity (P<0.05), with strain-specific colonization differences. *In vitro*, *dltD* deletion strains showed decreased biofilm viable cells (P<0.05), metabolic activity (P<0.01), and water-insoluble polysaccharides (P<0.01), associated with downregulated *gtfB* and *gtfC* expression (P<0.05), increased autolysis, and extracellular DNA (P<0.01). Acidogenicity and acid tolerance were impaired, associated with downregulated *dexA*, *fabM*, and *atpD* expression (P<0.05).

**Discussion:**

These findings confirmed that *dltD* deletion attenuates *SM* cariogenicity by disrupting biofilm EPS and acid metabolism, supporting *dltD* as a potential target for caries prevention.

## 1 Introduction

Dental caries has been classified by the World Health Organization as a priority noncommunicable disease requiring targeted prevention and management strategies (Global Oral Health Status Report, 2022). Recent epidemiological data indicate that approximately 2.3 billion individuals worldwide are affected by dental caries of varying severity (Bernabe et al., 2020). Notably, dental caries not only serves as the primary etiological factor for pulpitis and periapical diseases but also has been identified as a potential risk factor for multiple systemic conditions, including endocarditis (Hsu et al., 2022) and ischemic stroke (Yu et al., 2024). Given that the global population is aging and health care expenditures are increasing, the economic burden imposed by dental caries has emerged as a pressing challenge for health systems worldwide (Hugo et al., 2021).

The “ecological plaque hypothesis” posits that dental caries is essentially a dynamic process of oral microecological imbalance (Radaic and Kapila, 2021). Cariogenic bacterial communities possess acidogenic and aciduric properties. When cariogenic bacterial communities dominate the plaque microenvironment, the sustained acidic environment induces demineralization of hard dental tissues (Spatafora et al., 2024). This ultimately leads to the formation of carious lesions. Among the diverse array of cariogenic microorganisms, *Streptococcus mutans* (*SM*) is recognized as one of the key strains involved in the progression of dental caries. *SM* exhibits excellent biofilm formation ability, stable acid-producing and acid-tolerant characteristics, and a rapid environmental stress response (Shanmugam et al., 2020). It serves as a biomarker for the occurrence of dental caries (Li et al., 2021). Its complex virulence regulation network has become an important target in current ecological caries prevention research (Chen et al., 2022).

The *dltD* gene is located within the *dlt* operon and represents a conserved sequence among gram-positive bacteria (Neuhaus and Baddiley, 2003). Its primary function is to regulate the D-alanylation modification of the lipoteichoic acid (LTA) side chain in *SM* (Neuhaus and Baddiley, 2003). Specifically, the *dltD* gene facilitates the incorporation of positively charged D-alanine (D-Ala) into the LTA side chain (Wood et al., 2018). This process reduces the negative charge on the bacterial cell wall surface and is critical for sustaining membrane homeostasis (Chastain et al., 2025).

Castillo et al. reported that deleting the *dltD* gene in the *SM*UA159 resulted in a statistically significant reduction in the cariogenic capacity in a rat caries model(P<0.05), additionally, a reduced tolerance to hydrogen peroxide was observed in the *dltD* mutant strain compared with that in the wild-type under oxidative stress conditions (Castillo Pedraza et al., 2019). Midian et al. revealed disrupted biofilm architecture in the *SM*UA159 *dltD* mutant, characterized by microcolony-free zones in 3D structures (Castillo Pedraza et al., 2017). Compared with *SM*UA159, the *dltD* mutant strain showed no significant differences in extracellular DNA (eDNA) or water-insoluble extracellular polysaccharide (WIP) content in the 67 h and 115 h biofilms, notably, the water-soluble extracellular polysaccharide (WSP) content in the *dltD* mutant 67 h biofilm exhibited a marked increase (Castillo Pedraza et al., 2017). Findings from our research group indicate that deleting the *dltD* gene in *SM* 593—a highly cariogenic clinical strain of *SM*—leads to three key outcomes: enhanced bacterial autolysis, a loose and porous biofilm structure (Du et al., 2024), and diminished acid resistance (Du et al., 2022). Recently, we observed that the biofilm of *SM*UA159 also exhibits phenotypes of increased autolysis, reduced viable bacteria in the biofilm, and decreased metabolic activity of the biofilm, which is consistent with the phenotype of strain *SM* 593 (**Supplementary Files**). Although the *dltD* gene is relatively conserved in *SM*, whether it exerts the same influence on the cariogenicity of different strains remains unclear.​ Therefore, we intend to further compare the effect of the *dltD* gene on the cariogenicity of different strains using both *in vivo* and *in vitro* experiments, thereby providing a theoretical basis for *dltD* gene to serve as a target for caries prevention and treatment.

Therefore, in this study, *SM*UA159, highly cariogenic clinical strains of *SM* 593, and their corresponding *dltD* gene deletion strains were selected as the experimental strains. The aim of the present study was to compare the influence of the *dltD* gene on the cariogenic phenotypes of different *SM* strains, thereby providing a theoretical basis for the potential application of the *dltD* gene as a target in the prevention and treatment of dental caries.

## 2 Materials and methods

### 2.1 Bacterial strains and culture conditions

The bacterial strains and plasmids used in this study are listed in **Table 1**. *SM* 593 and *SM*UA159 strains were routinely cultured in brain-heart infusion (BHI) broth (OXIOD, UK) or BHI broth supplemented with 1% (w/v) sucrose (Difco, Sparks, MD, USA). Cultivation was performed at 37°C under anaerobic conditions consisting of 90% N_2_, 5% CO_2_, and 5% H_2_. *Escherichia coli* (*E. coli*) was grown aerobically in Luria-Bertani medium (Oxoid Ltd.) at 37°C. For selective cultivation, spectinomycin (Sigma-Aldrich, USA) was used at 1 mg/mL for the *SM*UA159 *dltD* deletion mutant and 100 μg/mL for *E. coli.* Chloromycetin (20 μg/mL Solarbio, China) was added when needed for the *SM*UA159 *dltD*-complemented strain and *E. coli* cultures.

**Table 1:** List of major strains and plasmids (Sp^r^: spectinomycin resistance; CHL^r^: chloromycetin resistance).

Strains or plasmids	Description	Source
*SM*UA159	Wild type	Laboratory preservation
*SM*UA159-Δ*dltD*	*SM*UA159 *dltD* dilatation mutant; Sp^r^	This study
*SM*UA159-Δ*dltD*-c	Complemented strain; CHL^r^	This study
*SM* 593	Wild type	Laboratory preservation
*SM* 593-Δ*dltD*	*SM* 593 *dltD* dilatation mutant; Sp^r^	Laboratory preservation
*SM* 593-Δ*dltD*-c	Complemented strain; CHL^r^	Laboratory preservation
pFW5	*E. coli-Streptococcus* shuttle expression vector; Sp^r^	Laboratory preservation
pFW5-*dltD*	for *dltD* knockout; Sp^r^	Previously constructed
pIB169	*E. coli-Streptococcus* shuttle expression vector; CHL^r^	Presented by Professor Huang
pIB169-*dltD*	for *dltD* complemented; CHL^r^	Previously constructed

In the rat caries model, we used blood agar to culture total bacteria and selective mitis salivarius bacitracin (MSB) to cultivate *SM*. The culture medium preparation methods were as follows. To make blood agar plates: Dissolve 7.4 g of BHI powder, 0.2 g of yeast extract, and 2.5 g of agar powder were dissolved in 200 mL of double-distilled water (ddH_2_O) under continuous stirring. The mixture was autoclave at 121°C for 15 min. After cooling to 45–55°C, the medium was aseptically supplemented with 5 mg/L hemin chloride, 1 mg/L vitamin K_1_, and 10 mL of sterile defibrinated sheep blood. The medium was mixed thoroughly and poured into Petri dishes for total bacterial cultivation. To make MSB Medium, 18 g of Mitis Salivarius agar was dissolved in 200 mL of ddH_2_O with agitation. The medium was sterilized by autoclaving at 121°C. Upon cooling to 45–55°C, one vial of S3401 (containing 2 mg of potassium tellurite) (Shandong Tuopu Biol-engineering Co., Ltd., China) and two vials of S0703 bacitracin (20 U/vial) (Shandong Tuopu Biol-engineering Co., Ltd., China) were added. The medium was then vortexed to homogenize and dispense into plates for the selective growth of *SM*.

### 2.2 Construction of the *SM*UA159-Δ*dltD* and *SM*UA159-Δ*dltD*-c strains

The *SM*UA159 *dltD* deletion strain (*SM*UA159-Δ*dltD*) was constructed following a previously described protocol (Du et al., 2024). Briefly, upstream and downstream homologous fragments of the *SM dltD* gene were inserted flanking the spectinomycin resistance gene in the pFW5 plasmid. Specifically, the upstream homologous fragment was cloned and inserted into the BamHI/HindIII restriction sites, and the downstream fragment was inserted into the NcoI/SmaI sites (Sangon Biotech, China). The recombinant pFW5 plasmid was then transformed into the wild-type *SM*UA159 strain. The spectinomycin resistance gene in pFW5 replaced the endogenous *dltD* gene in *SM*UA159 via homologous recombination. Deletion strains (Δ*dltD*) were selected on agar plates supplemented with spectinomycin. Successful construction of the *dltD* knockout strain was verified using polymerase chain reaction (PCR) and gene sequencing.

To construct the *SM*UA159 *dltD* complemented strain (*SM*UA159-Δ*dltD*-c), the full-length *dltD* gene fragment from *SM*UA159 was inserted into the EcoRI/BamHI restriction sites of the pIB169 plasmid (Sangon Biotech, China). The recombinant pIB169 plasmid was subsequently transformed into the *SM*UA159-Δ*dltD* strain. Positive clones of the complemented strain were isolated by selection on chloramphenicol-containing agar plates. Initial screening for *dltD* gene complementation was performed via PCR, and further validation was conducted using reverse transcription–quantitative PCR (RT–qPCR).

### 2.3 Colony-forming units

The CFU assay was used to detect the viable biomass. After the bacterial suspension was centrifuged, the pellet was rinsed three times with phosphate-buffered saline (PBS). An equal volume of PBS was then added to resuspend the bacterial cells. The cells were diluted 10^1–^10^10^ times, and each concentration was coated on BHI agar plate with 100 μL of bacterial solution. After 24 h of culture under anaerobic conditions, the cells were counted.

### 2.4 *In vivo* dental caries model

#### 2.4.1 Ethics approval

The animal experiments were approved by the Animal Ethics Committee of Fujian Medical University (Approval No.: IACUC FJMU 2023-0236).

#### 2.4.2 Infection and maintenance of rats

All animal experimental protocols were reviewed and approved by the Institutional Animal Care and Use Committee of Fujian Medical University.

Sprague-Dawley (SD) rats were housed in specific pathogen-free (SPF) facilities at the Animal Experiment Center of Fujian Medical University. The environmental conditions were strictly controlled to maintain physiological stability: the ambient temperature was maintained at 25 ± 2°C, the relative humidity was maintained at 55 ± 5%, and a 12-hour light/dark cycle was used to simulate natural circadian rhythms. Throughout the entire experimental period, the rats had ad libitum access to autoclaved drinking water and standard rodent chow to eliminate nutritional variability as a confounding factor. The experimental cohort was randomly divided into seven groups (n = 6 rats per group). The groups were designated as follows: (1) control group (no bacterial inoculation); (2) *SM*UA159 group (inoculated with *SM*UA159 wild-type strain); (3) *SM*UA159-Δ*dltD* group (inoculated with *SM*UA159-Δ*dltD*); (4) *SM*UA159-Δ*dltD*-c group (inoculated with *SM*UA159-Δ*dltD*-c); (5) *SM* 593 group (inoculated with *SM* 593 wild-type strain); (6) *SM* 593-Δ*dltD* group (inoculated with *SM* 593-Δ*dltD*); and(7) *SM* 593-Δ*dltD*-c group (inoculated with *SM* 593-Δ*dltD*-c).

Before microbial colonization, the SD rats were given drinking water supplemented with ampicillin (1 g/L) for 3 consecutive days; this was followed by an antibiotic washout period using sterile standard water to eliminate residual ampicillin (Hsiao et al., 2019). To confirm the absence of preexisting *SM* colonization before bacterial inoculation, mucosal swabs were collected from the oral cavities of the rats, streaked onto MSB plates, and incubated anaerobically at 37°C for 48 h. To prepare the preparation of bacterial inoculum, revived *SM* strains (including the wild-type strain, Δ*dltD*, and complemented strains) were subcultured in brain-heart infusion (BHI) broth at a 1:20 dilution and cultured to the mid-exponential growth phase. The bacterial cultures were then centrifuged at 4,000 ×g for 5 minutes, after which and the pellets were washed twice with PBS. Finally, the pellets were resuspended in PBS to adjust the optical density at OD_600nm_=0.5. Bacterial suspensions (for experimental groups) or PBS (for negative controls) were administered to the mandibular molars of rats via oral swabbing, with this application repeated daily for a consecutive 4-day period. After the initial inoculation, the diet of the rats was changed to Diet 2000 (supplemented with 56% sucrose; Trophic Animal Feed High-tech Co., Ltd, China), and the rats they were provided with ddH_2_O supplemented with 5% sucrose for 35 days. On day 5 after the initial inoculation, mucosal swabs were collected and cultured to verify the colonization of *SM*. To monitor the general physiological status of the rats and rule out potential confounding effects of nutritional deficiency or disease on experimental outcomes, body weight measurements were conducted at 5-day intervals throughout the modeling period.

#### 2.4.3 Viable biomass assay

Following successful modeling, the rats were anesthetized via intraperitoneal injection of pentobarbital sodium. The mandibular bones were dissected from each rat. For microbial detachment, the dissected mandibular specimens were transferred to sterile PBS and subjected to ultrasonic agitation. The resulting bacterial suspensions from individual mandibles were serially diluted in 10-fold increments using sterile PBS. From each dilution, a 100 μL aliquot was aseptically plated onto two types of culture media for differential microbial enumeration: (1) a blood agar plate, used for quantifying the total cultivable bacterial population; and (2) MSB for the specific enumeration of *SM*. Following inoculation, blood agar plates were incubated aerobically at 37°C, whereas the MSB plates were cultured in an anaerobic environment (90% N_2_, 5% CO_2_, and 5% H_2_) to optimize *SM* growth. After 48 hours of incubation, CFUs on each plate were counted.

#### 2.4.4 Caries scores assay

After ultrasonic treatment, the mandibles were rinsed twice with PBS and fixed in 4% paraformaldehyde. The samples were stained overnight in 0.4% (w/v) ammonium purpurate. After staining, the molars were subjected to mesiodistal sectioning using a low-speed precision saw, exposing the pulp chambers for magnified inspection. Stereomicroscopy was employed to assess the caries score. Caries severity was graded per Keyes criteria (KEYES, 1958): E (enamel-limited), Ds (≤1/4 dentin), Dm (1/4–3/4 dentin), and Dx (>3/4 dentin). Two blinded independent evaluators performed scoring; discrepancies exceeding one severity grade required third-party arbitration, with final scores derived from consensus between the two closest assessments.

### 2.5 Adhesion ability

The glass tube method was employed for the detection of bacterial adhesion ability (Wu et al., 2022). An overnight-cultured bacterial suspension was inoculated into Tube A at a 1:20 (v/v) ratio with BHI medium containing 1% sucrose. The inoculated tube A was incubated anaerobically at 37°C for 24 h with a 30° inclination. After incubation, Tube A was gently inverted 3 times, and the resulting suspension was transferred to Tube B (as nonadherent bacteria). The bacterial suspension in Tube B was centrifuged to discard the supernatant, rinsed twice with PBS, and finally resuspended in an equal volume of PBS. Subsequently An equal volume of PBS was subsequently added to Tube A (as adherent bacteria), followed by vortexing until all the adherent bacteria were completely detached. The OD_600nm_ values of all bacterial suspensions was measured. The percentage of adherent bacteria was calculated using the formula: adherent bacteria percentage = (OD_600nm_ of adherent bacteria/(OD_600_ of adherent bacteria + OD_600_ of nonadherent bacteria)) × 100%.

### 2.6 Biofilm formation

Biofilms were cultivated in 24-well plates, where each well contained 1 mL of BHI broth supplemented with 1% sucrose. Before inoculation, the bacterial strain was cultured to the mid-exponential growth phase, and its cell suspension was adjusted to an OD_600_nm__ ≈ 0.2. This standardized suspension was then diluted at a ratio of 1:20 and used to inoculate all the wells. Following inoculation, the plates were placed in an anaerobic environment and incubated at 37°C for 24 hours.

### 2.7 Extracellular polysaccharide production assay

To analyze the causes of biofilm structure changes in the *dltD* mutant strain, we compared the contents of WIP and WSP between the mutant strain and the wild-type strain, while observing the distribution of extracellular polysaccharides (EPS) using confocal laser scanning microscopy (CLSM).​ The contents of water-insoluble extracellular polysaccharides (WIP) and water-soluble extracellular polysaccharides (WSP) synthesized by experimental strain biofilms were quantified via the anthrone-sulfuric acid method (Wu et al., 2022). After biofilm maturation, the supernatants were discarded, and the biofilms were rinsed with PBS. The biofilms were then resuspended in an equal volume of PBS and centrifuged at 3000 × g for 20 min at room temperature. The resulting supernatant was collected, and this washing–centrifugation step was repeated twice to ensure complete removal of WSP; all the supernatants from these steps were pooled for subsequent analysis. Following WSP removal, WIP in the remaining biofilms was extracted by washing three times with 0.4 M NaOH, and all the supernatants from the extractions were collected. Both WSP and WIP samples were quantified using the anthrone method: 300 μL of anthrone and 3 mL of 98% sulfuric acid were added to each sample, followed by a 10 min incubation before measuring the OD_630nm_ was measured. A calibration curve was generated using pure sucrose, and all measurements were performed in triplicate to calculate the mean values and standard deviations.

Biofilm EPS distribution was observed by CLSM. Alexa Fluor™ 647 (Thermo Fisher) was added to the culture medium during biofilm development. After the experimental strains formed biofilms on circular glass coverslips, the samples specimens were aseptically rinsed three times with PBS to remove planktonic cells. They were then fixed in 2.5% glutaraldehyde solution (light-protected) at 4°C for 2 h. The specimens were then stained with SYTO9 nucleic acid dye for 15 min in the dark to label live cells, followed by three PBS washes to remove unbound dye. The processed samples were mounted on glass slides using anti-fade medium and analyzed via CLSM (Leica TCS SP8). Three random fields per sample were imaged with a 63× oil-immersion objective (z-stack interval: 0.5 μm). 3D reconstruction and biofilm thickness analysis were performed using ImageJ. The fluorescence intensity was quantified via ImageJ’s threshold-based particle analysis, with values normalized to background signals.

### 2.8 Biofilm protein content assay

Proteins are important components of the biofilm matrix and influence biofilm structure. To investigate the causes of biofilm structure changes in the *dltD* mutant strain, we compared the content of extracellular protein contents between the mutant strain and the wild-type strain. Following biofilm maturation, the specimens were gently rinsed three times with PBS. For matrix protein extraction, the biofilms were resuspended in an equal volume of PBS and subjected to dual centrifugation cycles (4,000 ×g, 20 min, 4°C). The pooled supernatant fractions were collected as test samples for subsequent analysis. Fresh BCA working reagent was prepared by mixing the Beyo BCA Rapid Protein Assay Kit components according to the manufacturer’s specifications, followed by vortex homogenization and 10 min equilibration at ambient temperature. Aliquots (20 μL) of test samples were dispensed into a 96-well microplate, with each well receiving 200 μL of the BCA working solution. The plate was incubated under light-protected conditions at 37°C for 30 min. The OD_562nm_ was measured using a microplate reader, with PBS-treated blank wells serving as an optical reference. Protein concentrations were calculated using a standard curve generated from serial BSA dilutions.

### 2.9 pH drop assay

Acid-producing capacity is among the important cariogenic virulence factors of *SM*. We measured the biofilm acid-producing capacity of the *dltD* mutant strain and wild-type strains using a pH drop assay (Wu et al., 2022). Following biofilm maturation, the supernatants from the experimental groups were aseptically aspirated and centrifuged at 4000 × g for 5 min at ambient temperature to pellet residual cellular debris. The clarified supernatant fractions were promptly transferred to sterile microtubes, and their pH values were measured using a digital pH meter (Thermo Fisher, USA).

### 2.10 Intracellular polysaccharide assay

As an endogenous carbohydrate of *SM*, IPS serves as one of the carbohydrate sources. It can be metabolized via the glycolytic system, continuously supply energy to bacteria, and extend the duration acid-producing in cells (Costa Oliveira et al., 2021). To further investigate the reason for the decreased acid-producing capacity of the *dltD* mutant strain, we detected the IPS content. Mature biofilms were rinsed three times with PBS to remove planktonic cells and then resuspended in an equal volume of ddH_2_O. The suspensions were heat-treated at 100°C for 5 min, followed by centrifugation at 4,000×g for 10 min at 4°C; the supernatants were subsequently discarded. The resulting pellets were then washed twice with ice-cold ddH_2_O and resuspended in 1 mL of ddH_2_O. To each sample, 0.3 mL of 5.3 M KOH was added, and the mixtures were incubated at 100°C for 90 min. After cooling to 25°C, 0.3 mL of 5.3 M HCl and 1.0 mL of 1.0 M potassium phosphate buffer (pH 7.0) were mixed by vortexing to ensure homogeneity. Afterward, 0.6 mL of fresh iodine reagent was introduced into each tube. The vortex-mixed samples were incubated in the dark for 10 min, after which 200 μL aliquots were then analyzed for their OD_520nm_ values using a microplate reader (Molecular Devices SpectraMax^®^). Purified water served as the blank, and a standard curve, prepared from a 1 mg/mL glycogen stock (Sigma), was used to calculate the IPS concentration in the samples.

### 2.11 Lactate dehydrogenase activity assay

LDH is responsible for converting pyruvate into lactic acid and serves as one of the key enzymes in the glycolytic pathway of *SM* (Feng et al., 2018). To investigate the cause of the difference in acid production between the *dltD* mutant and the wild-type strains, we detected the LDH enzyme activity. An overnight bacterial culture was inoculated into BHI broth at a 1:20 (v/v) ratio and incubated until reaching the mid-exponential growth phase was reached. The bacterial suspension was then centrifuged at 4000 rpm for 5 min; the supernatant was discarded, and the bacterial pellet was washed twice with PBS. The pellet was resuspended such that the OD_600_nm__ ≈ 0.2, which corresponds to approximately 10^8^ CFU/mL. Following the instructions provided for the LDH kit (Solarbio, China), the extraction buffer was added to the bacterial suspension, and the mixture was sonicated on ice (200 W, 3 s on, 10 s off, 30 cycles). The sonicated sample was centrifuged at 8000 × g for 10 min, and the resulting supernatant was collected and kept on ice until subsequent analysis.

### 2.12 Acid tolerance assay

Biofilms were established as previously described. After aspirating the supernatant medium was aspirated, the samples were rinsed three times with PBS. For treatment, the direct acidification group was supplemented with an equal volume of BHI broth, whereas the preacidification group received the same volume of BHI broth adjusted to pH 5.5. Both groups were anaerobically incubated for 3 h. After incubation, the supernatant was aspirated, and the samples were rinsed three times with PBS to eliminate planktonic bacteria. Each group was then exposed to 0.1 M glycine buffer (pH 3.5) for 15 min, 30 min, or 45 min. Serial dilutions of the samples were prepared and plated on agar plates; CFU counts were determined following 48 h of anaerobic incubation.

### 2.13 Laurdan staining

SM can increase its survival ability under acidic conditions by increasing membrane fluidity (Boisen et al., 2021). In this study, the cell membrane fluidity of the strain under different pH conditions was detected using a Laurdan fluorescent probe. Laurdan is a fluorescent probe that can be used to study cell membrane fluidity. The membrane fluidity was compared using the generalized polarization (GP) value. The GP value ranges from −1 to +1, and a higher GP value indicates poorer membrane fluidity (Scheinpflug et al., 2017). Strains cultured to mid-exponential phase in pH 5.5 BHI broth were centrifuged to remove the medium, washed with deionized water, and resuspended in 200 μL of 10 μM Laurdan (MedChemExpress, China). The suspension was incubated at 37°C for 1.5 h. Then, the fluorescence intensity was measured using a microplate reader at λex = 350 nm and λem = 440/490 nm. The membrane fluidity was compared by calculating the GP using the formula: GP = (I440 − I490)/(I440 + I490).

### 2.14 RNA isolation and reverse-transcription quantitative PCR

RNA isolation was performed as previously described (Huang et al., 2022). Total RNA was extracted following the manufacturer’s instructions (Qiagen, Valencia), and genomic DNA contamination was removed using a PrimeScript™ RT reagent Kit with gDNA Eraser (Takara Bio, Otsu, Japan), which was also used for cDNA synthesis according to the manufacturer’s protocol. For gene expression analysis, 24 h biofilms were collected from six-well plates, treated with RNA protect Bacteria Reagent (Qiagen, Valencia, CA) to stabilize the RNA, and stored at −80°C. Total RNA extraction and cDNA synthesis were performed as described above. Quantitative real-time PCR (qPCR) was conducted in triplicate using 96-well plates (Sangon Biotech, Shanghai, China) with SYBR^®^ Premix Ex Taq™ (Tli RNaseH Plus) on a LightCycler 480 instrument (Roche, Basel, Switzerland). Gene-specific primers (listed in **Table 2**) were used to amplify biofilm formation-related genes (*gtfB*, *gtfC*, *gtfD*, *gbpA*, *gbpB*, *dexA*), autolysin-related genes (*atlE*), *dlt* operon-related genes (*dltA*, *dltB*, *dltC*), IPS-related genes (*glgA*, *glgB*, *glgC*, *glgD*), and acid tolerance-related genes (*atpD*, *fabM*). The expression levels were normalized to those of the 16S rRNA reference gene and calculated using the 2^-ΔΔCT^ method, with the 2^-ΔCT^ method applied for target gene normalization to the respective references.

**Table 2:** Nucleotide sequences of primers were used in this study.

Primer	Sequence (5’-3’)	Description	Reference
*gtfB*-F	5’-TGCCGCAGTCCCTTCTTATTC-3’	Insoluble glucan production	This work
*gtfB*-R	5’-GCCATGTATTGCCCGTCATCT-3’		
*gtfC*-F	5’-GTGCGCTACACCAATGACAGAG-3’	Insoluble and soluble glucan production	
*gtfC*-R	5’-GCCTACTGGAACCCAAACACCTA-3’		
*gtfD*-F	5’-TACCTTGGGCACCACAACACT-3’	Soluble glucan production	
*gtfD*-R	5’-TGCCGCCTTATCATCCTCACT-3’		
*gbpA*-F	5’-GCCGAGCGTATCAGTACAGTTGAG-3’	Glucan-binding protein	
*gbpA*-R	5’-CCGTCATCAGGCACAGAACCAC-3’		
*gbpB*-F	5’-CAGCAGCGGCAGGATATAGAGTTG-3’	Glucan-binding protein	
*gbpB*-R	5’-ACGTGTCCATAACCGCCATCATTC-3’		
*atlE*-F	5’-AGCTGGTCCCAAAGGAAATC-3’	Autolysin	(Khan et al., 2017)
*atlE*-R	5’-GCCTGTGCCCAATAATCATC-3’		
*dltA*-F	5’-CTGATTCGCTTGCAGCTCATCTTG-3’	D-alanine-poly (phosphoribitol) ligase subunit	(Florez Salamanca and Klein, 2018)
*dltA*-R	5’-CCAGCATGGCATATTCCTGTCCTC-3’		
*dltB*-F	5’-ACTCAACGACGCTTGGCTTC-3’	D-alanyl-lipoteichoic acid biosynthesis protein	
*dltB*-R	5’-CCTAACGCTCTTGTCCAGCC-3’		
*dltC*-F	5’-ATGCAGGTGTTTTAGATAGCATGG-3’	D-alanine-poly(phosphoribitol) ligase subunit	
*dltC*-R	5’-TTGGCTGTATTCCAATCATCACGA-3’		
*glgA*-F	5’-ACGATCTGCATAGAGCACCG-3’	Glycogen synthase	(Costa Oliveira et al., 2021)
*glgA*-R	5’-TGGAGTTGGTGATGAGCGTT-3’		
*glgB*-F	5’-GTAAAGTGACCAGGCACCCA-3’	1,4-alpha-glucan branching protein	
*glgB*-R	5’-GGGCTTCTTTGCCTTTGAGC-3’		
*glgC*-F	5’-TCAACAAGCATGGTCCGTAA-3’	Glucose-1-phosphate adenylyltransferae	
*glgC*-R	5’-TCCAATGACCGTATCGTTGA-3’		
*glgD*-F	5’-TGGCAGTTGCGCGAAATAAT-3’	Glucose-1-phosphate adenylyltransferae subunit	
*glgD*-R	5’-AGCGACCTATTTTGCAGTGGA-3’		
*dexA*-F	5’-TATTTTAGAGCAGGGCAATCG-3’	Dextranase	(Florez Salamanca and Klein, 2018)
*dexA*-R	5’-AACCTCCAATAGCAGCATAAC-3’		
*atpD*-F	5’-GGCGACAAGTCTCAAAGAATTG-3’	F0F1 ATP synthase subunit beta	This work
*atpD*-R	5’-AACCATCAGTTGACTCCATAGC-3’		
*fabM*-F	5’-TAATGGTGTGGCGACTTTAAC-3’	Trans-2-decenoyl-ACP isomerase	
*fabM*-R	5’-AACAGCTCTTTGCATCTCAACC-3’		
16S-F	5’-AGCGTTGTCCGGATTTATTG-3’	House-keeping gene	(Huang et al., 2022)
16S-R	5’-CTACGCATTTCACCGCTACA-3’		

### 2.15 Statistical analysis

All experiments were repeated three times, with three samples per group. All values are expressed as the mean ± standard error. Statistical data were analyzed using ANOVA followed by Tukey’s multiple comparison tests in GraphPad Prism 8.0. Two-sided p-values less than 0.05 were considered to indicate statistical significance.

## 3 Results:

### 3.1 *dltD* deletion significantly attenuates the cariogenicity of *Streptococcus mutans*

**Figure 1:**
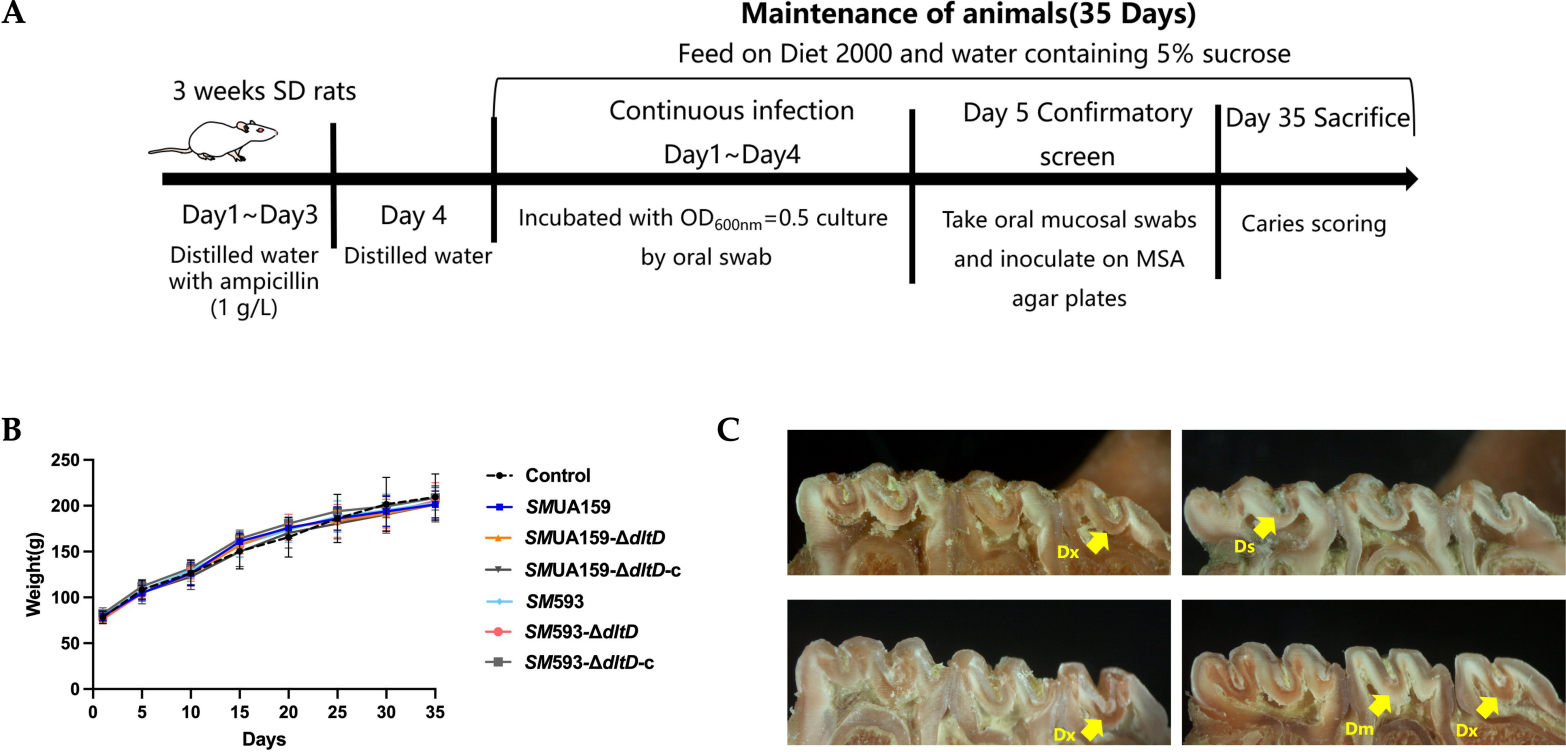
**(A)** Process of rat caries model; **(B)** Weight curve (n=6, the data is mean ± SD); **(C)** Sulcal caries under stereomicroscope (Ds: slight dentinal, all lesions in which the various processes have caused slight separation of the dentin from the overlying enamel up to and including involvement of approximately one-fourth of the dentin between the enamel and pulp chamber; Dm: moderate dentinal, dentinal penetration between one-fourth and three-fourths of the dentin between the enamel and pulp chamber; Dx: extensive dentinal, dentinal penetration beyond the three-fourth of the dentin between the enamel and pulp chamber).

The rat caries model was successfully established as illustrated in **Figure 1A**. Throughout the entire experimental period, all groups exhibited comparable body weight gain, and no statistically significant differences were observed (**Figure 1B**).

**Figure 2:**
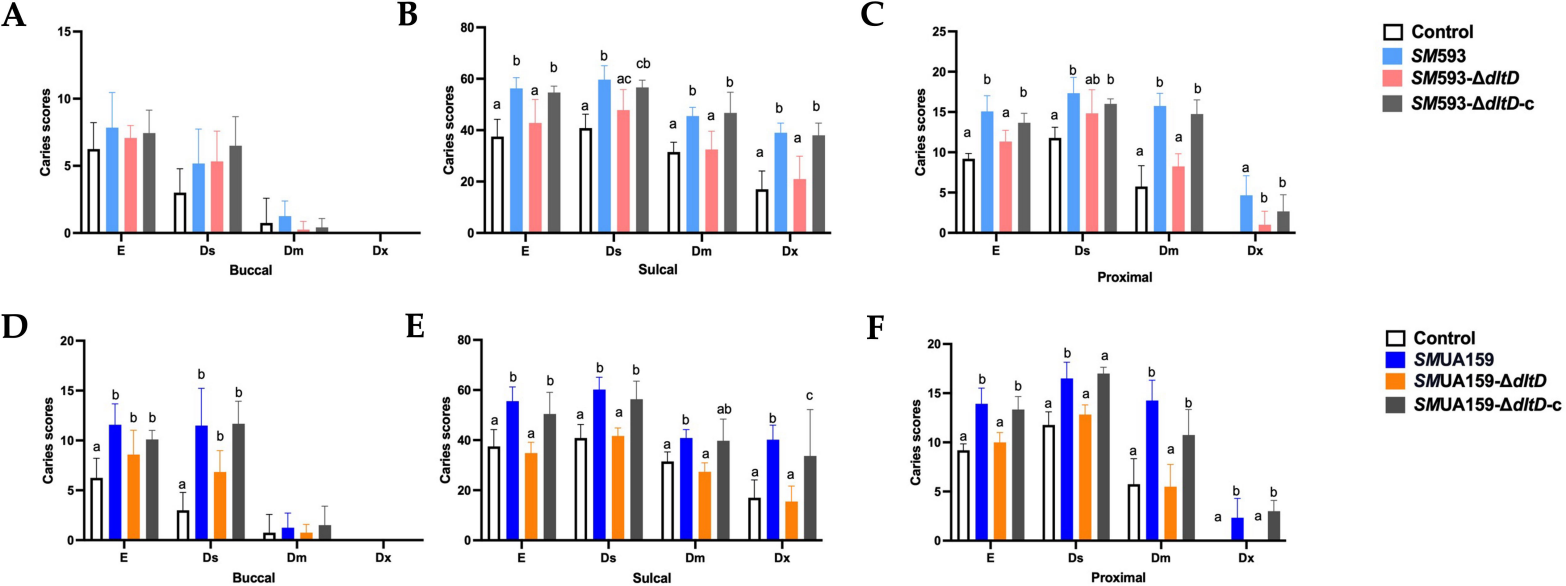
**(A)***SM* 593, *SM* 593-Δ*dltD*, *SM* 593-Δ*dltD*-c buccal caries scores; **(B)***SM* 593, *SM* 593-Δ*dltD*, *SM* 593-Δ*dltD*-c sulcal caries scores; **(C)***SM* 593, *SM* 593-Δ*dltD*, *SM* 593-Δ*dltD*-c proximal caries scores; **(D)***SM*UA159, *SM*UA159Δ*dltD*, *SM*UA159Δ*dltD*-c buccal caries scores; **(E)***SM*UA159, *SM*UA159-Δ*dltD*, *SM*UA159-Δ*dltD*-c sulcal caries scores; **(F)***SM*UA159, *SM*UA159-Δ*dltD*, *SM*UA159-Δ*dltD*-c proximal caries scores. (n=6, the data are mean ± SD, same lowercase letters in the same bar chart indicate no statistical difference, and different lowercase letters indicate statistical difference, P<0.05).

Caries scoring revealed that smooth surface caries scores of rats infected with *dltD* deletion strains (*SM* 593-Δ*dltD* and *SM*UA159-Δ*dltD*) showed a non-significant downward trend in smooth surface caries scores compared with those infected with wild-type strains (*SM* 593 and *SM*UA159). No caries lesions extending beyond 3/4 of the dentin thickness were detected in any group (**Figures 2A, D**). However, significant reductions (P < 0.05) in caries severity were observed for both sulcal caries and proximal surface caries in the mutant (**Figures 2B, C, E, F**).

**Figure 3:**
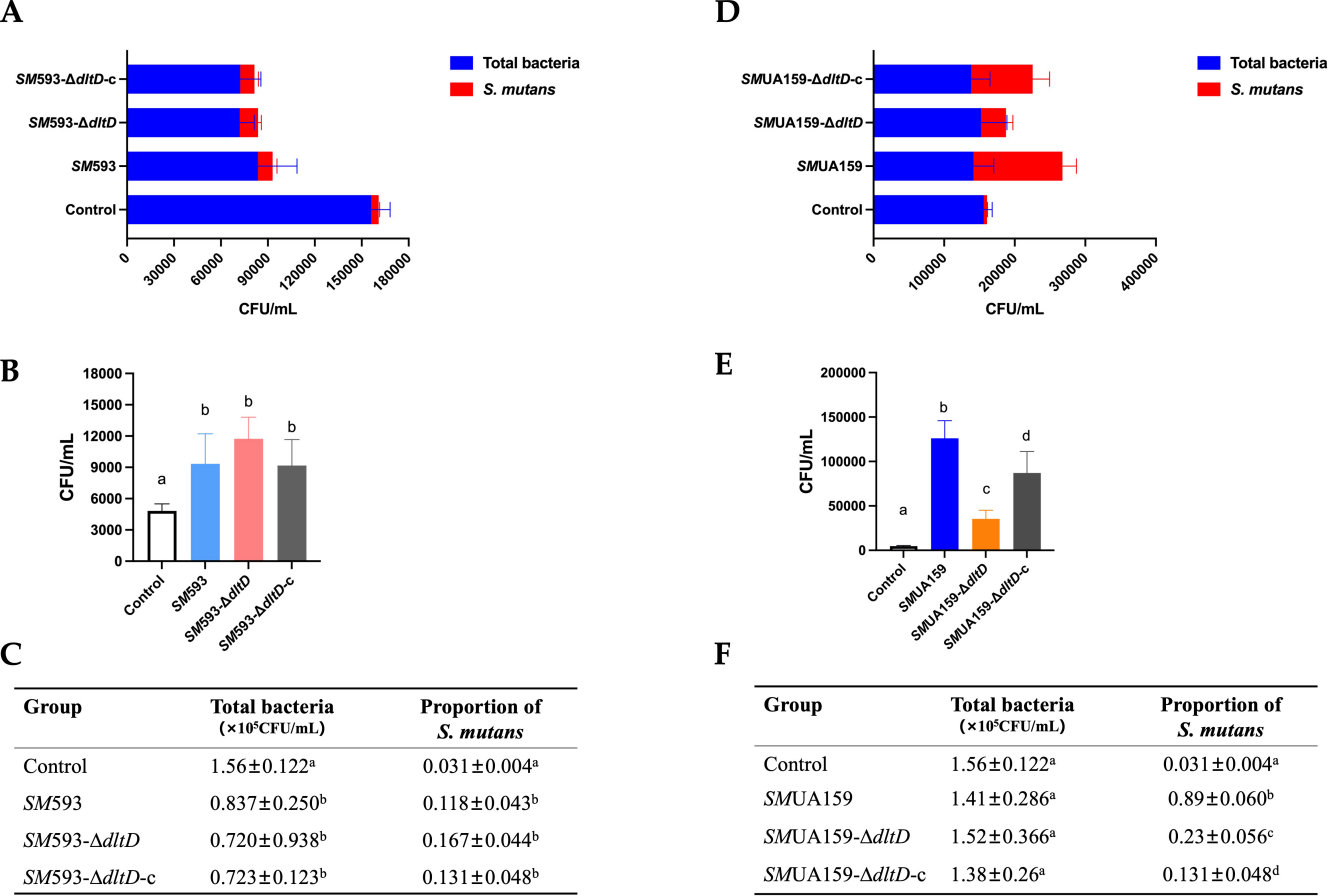
**(A, D)***SM* proportion of rat mandibular molars after sacrifice. **(B, E)***SM* CFU counting of rat mandibular tooth surface after sacrifice; **(C, F)** Total bacteria CFU counting and *SM* proportion. (n=6, the data are mean ± SD, same lowercase letters in the same bar chart indicate no statistical difference, and different lowercase letters indicate statistical difference, P<0.05).

Colonization Analysis:

For *SM* 593-derived strains:

Compared with wild-type *SM* 593-infected rats, rats infected with *SM* 593-Δ*dltD* exhibited comparable *SM* colonization levels on mandibular molars (**Figure 3B**), *SM* proportions (**Figures 3A, C**), and total bacterial loads (**Figures 3A, C**). Compared with the control group, both infected groups (*SM* 593 and *SM* 593-Δ*dltD*) demonstrated significantly reduced total bacterial loads (P < 0.05; **Figures 3A, C**).

For *SM*UA159-derived strains:

Compared with wild-type *SM*UA159 infection, infection with *SM*UA159-Δ*dltD* resulted in significantly reduced *SM* colonization of mandibular molars (**Figure 3E**) and a decreased *SM* plaque proportion (**Figures 3D, F**). Compared with the controls, both the *SM*UA159 and *SM*UA159-Δ*dltD* groups presented increased *SM* proportions (P < 0.05; **Figures 3D, F**), with no significant difference in total bacterial load (**Figures 3D, F**).

**Figure 4:**
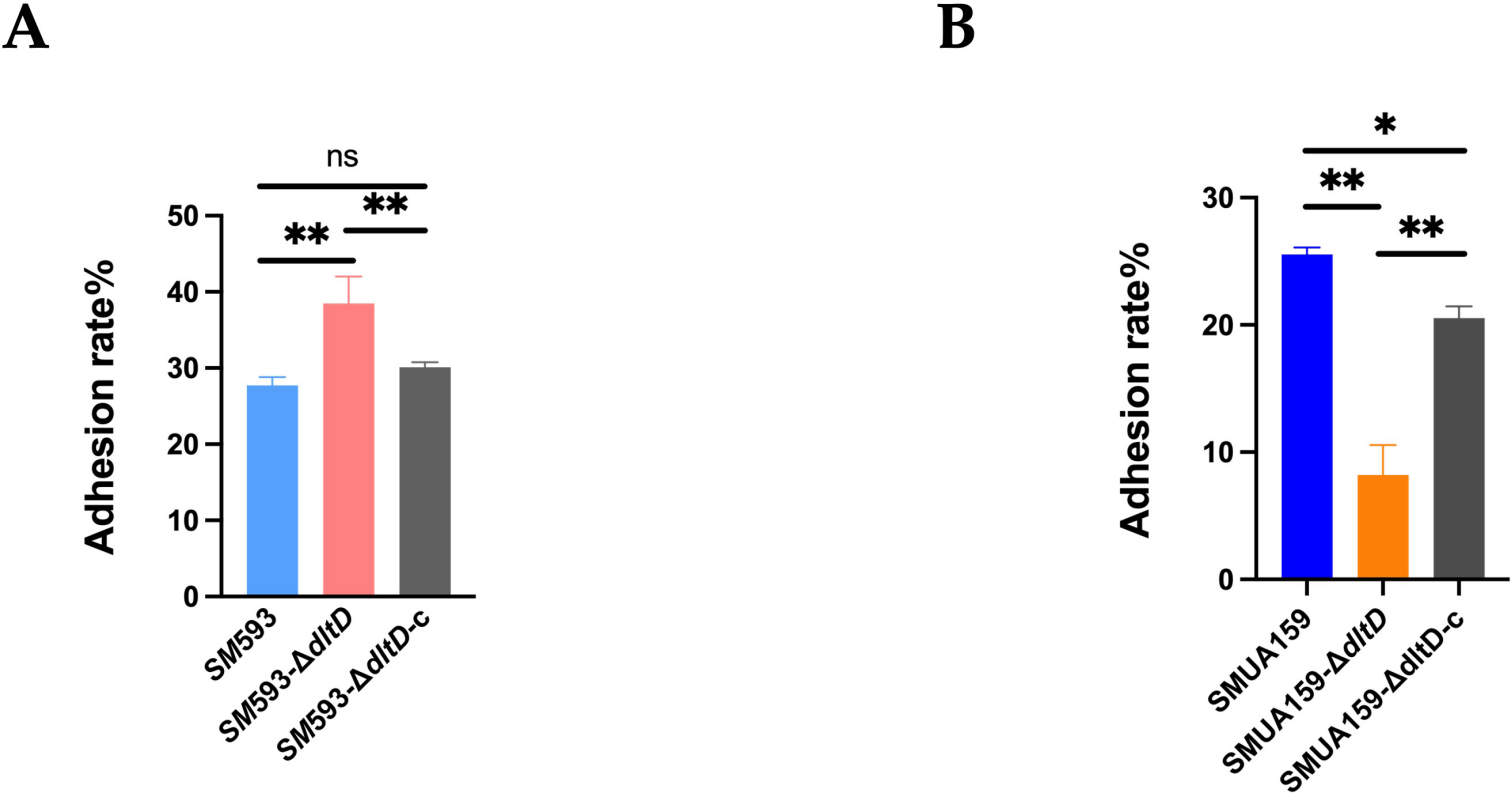
**(A)***SM* 593, *SM* 593-Δ*dltD*, *SM* 593-Δ*dltD*-c adhesion ratio; **(B)***SM*UA159, *SM*UA159-Δ*dltD*, *SM*UA159-Δ*dltD*-c adhesion ratio. (n=3, Data are presented as the mean ± SD, *P<0.05, **P <0.01).

*In vitro* adhesion assays revealed that compared with the adhesion of the wild-type strains, the adhesion of *SM* 593-Δ*dltD* to the glass interface was significantly greater (P<0.01; **Figure 4A**), whereas the adhesion of *SM*UA159-Δ*dltD* was significantly lower (P<0.01; **Figure 4B**).

### 3.2 *dltD* deletion alters extracellular matrix composition in *Streptococcus mutans*

**Figure 5:**
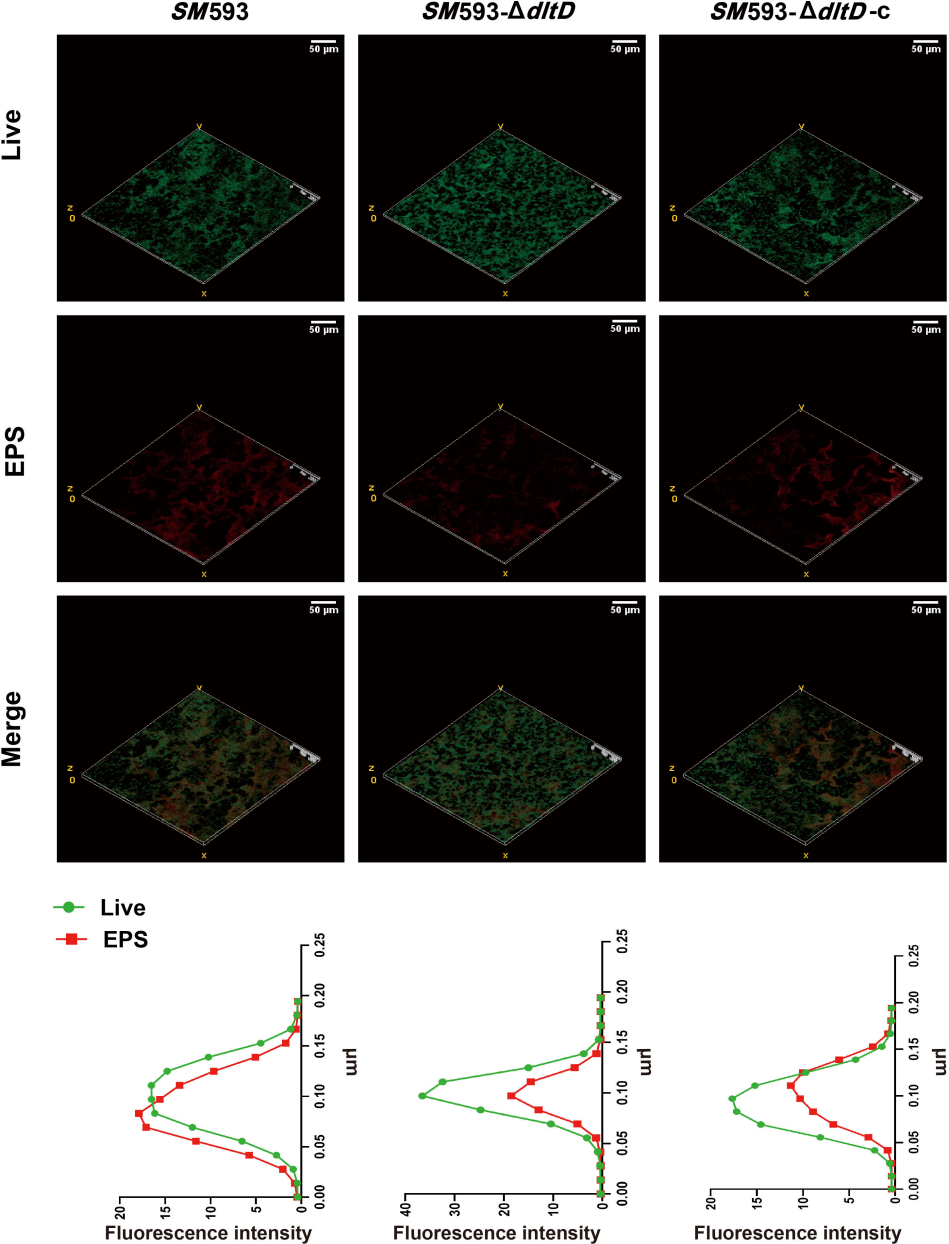
CLSM image of biofilm, green fluorescence represents living bacteria, and red fluorescence represents EPS.

**Figure 6:**
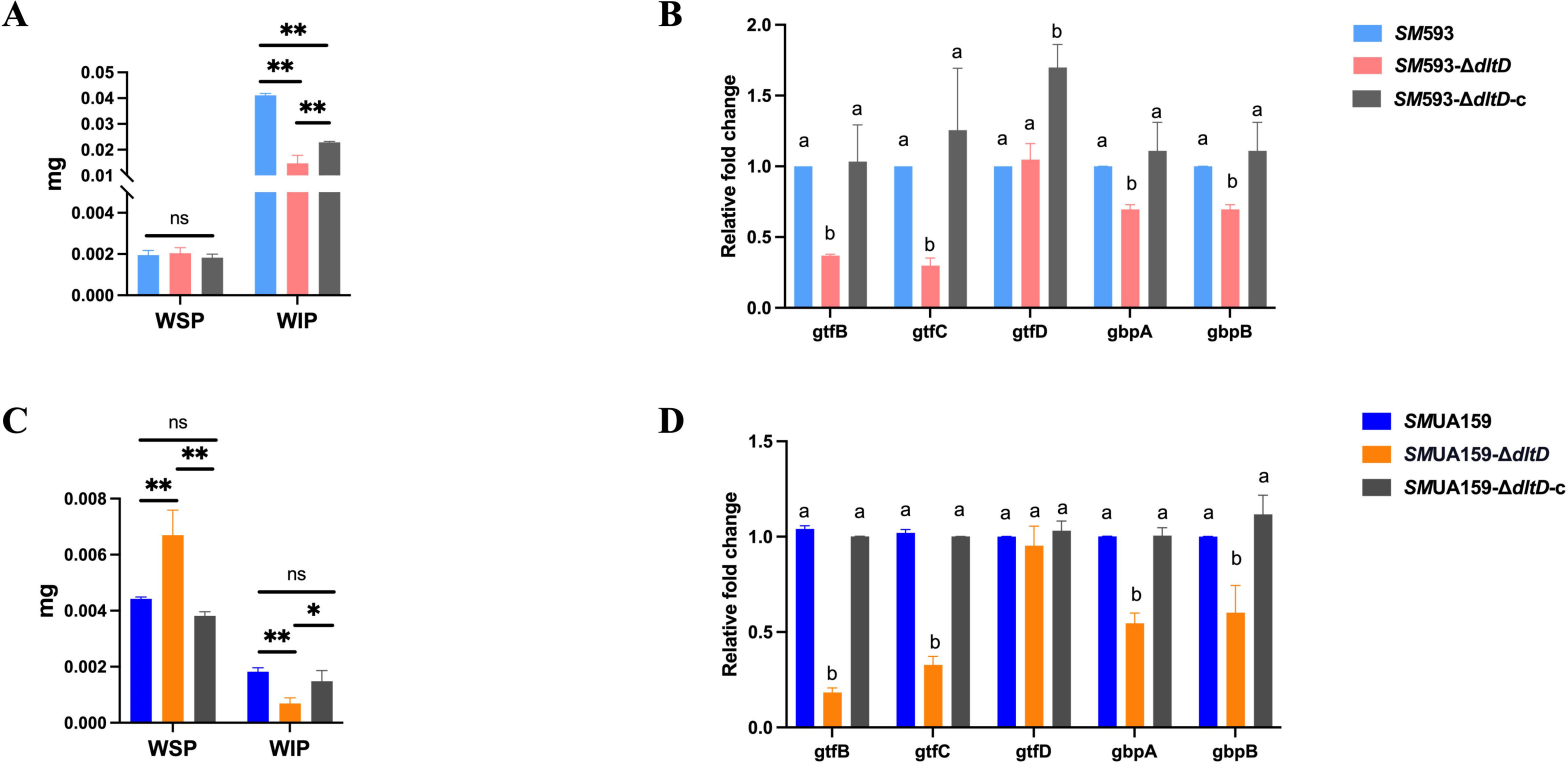
**(A)***SM* 593, *SM* 593-Δ*dltD*, *SM* 593-Δ*dltD*-c extracellular polysaccharide quantitative detection in biofilm: WSP was water-soluble extracellular polysaccharide, and WIP was water-insoluble extracellular polysaccharide; **(B)***SM* 593, *SM* 593-Δ*dltD*, *SM* 593-Δ*dltD*-c RT-qPCR for biofilm formation-related genes; **(C)***SM*UA159, *SM*UA159-Δ*dltD*, *SM*UA159-Δ*dltD*-c extracellular polysaccharide quantitative detection in biofilm; **(D)***SM*UA159, *SM*UA159-Δ*dltD*, *SM*UA159-Δ*dltD*-c RT-qPCR for biofilm formation-related genes. (n=3, Data are presented as the mean ± SD, *P<0.05, **P<0.01, same lowercase letters in the same bar chart indicate no statistical difference, and different lowercase letters indicate statistical difference, P<0.05).

CLSM revealed a marked reduction in red fluorescence intensity (representing EPS) in the *dltD* mutant relative to the wild-type strains (**Figure 5**). EPS quantification revealed no significant change in water-soluble polysaccharide (WSP) content (**Figure 6A**) but did reveal a significant decrease in water-insoluble polysaccharide (WIP) content (P<0.01; **Figure 6A**) in the *SM* 593-Δ*dltD* biofilm compared with the wild-type strains. However, compared with the wild-type strains, the *SM*UA159-Δ*dltD* biofilm exhibited a significant increase in WSP content (P<0.01; **Figure 6C**) and a significant decrease in WIP content (P<0.01; **Figure 6C**).

In *SM*, *gtfB* is responsible for WIP synthesis, *gtfC* is responsible for both WSP and WIP synthesis, *gtfD* is responsible for WSP synthesis, and *gbpA*/*gbpB* is responsible for anchoring EPS to *SM* cells (Matsumoto-Nakano, 2018; Zhang et al., 2021). RT–qPCR analysis revealed significantly downregulated expression levels of *gtfB*, *gtfC*, *gbpA*, and *gbpB* (P<0.05) in the *dltD* mutant, with no significant change in *gtfD* expression compared with that in the wild-type strains (**Figures 6B, D**).

**Figure 7:**
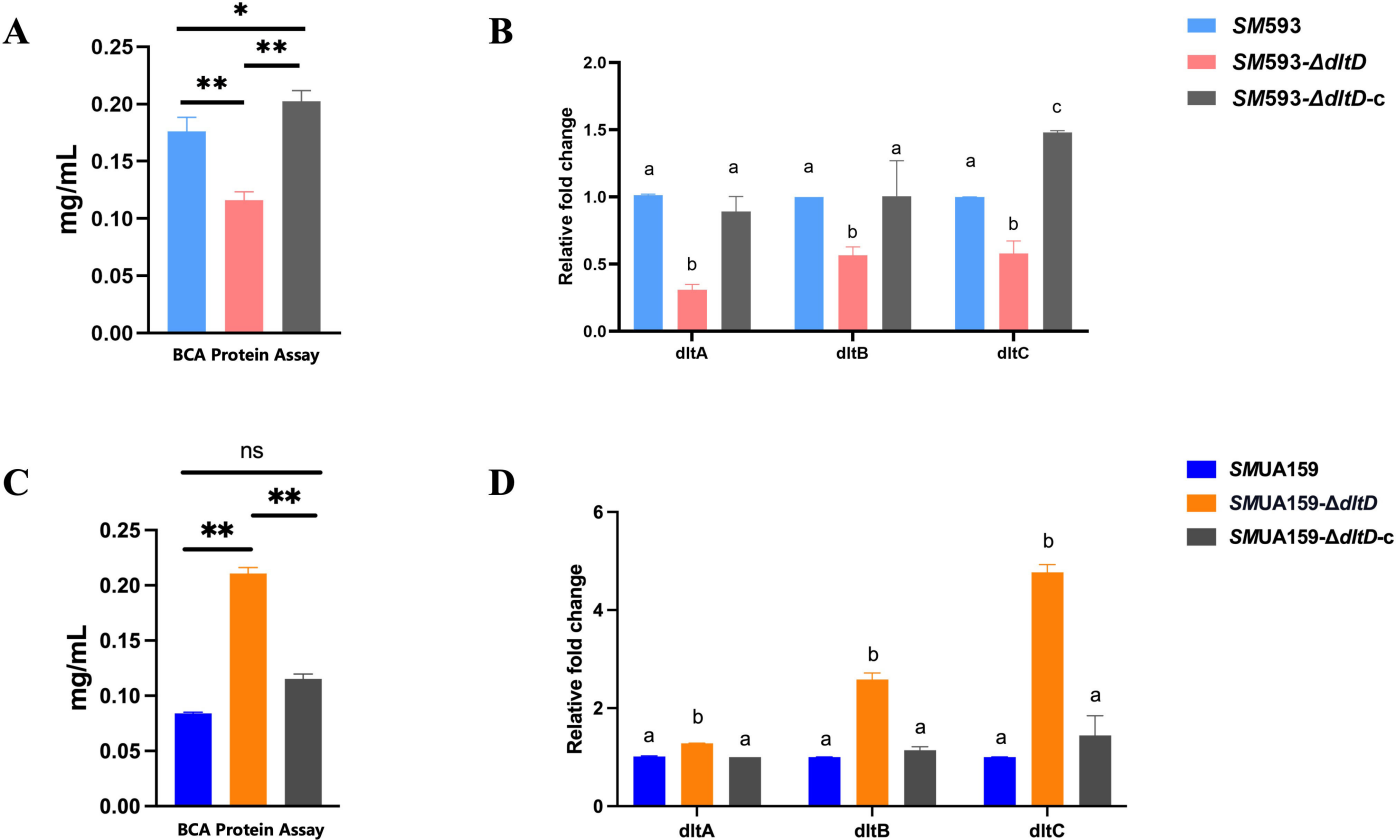
**(A)***SM* 593, *SM* 593-Δ*dltD*, *SM* 593-Δ*dltD*-c protein content; **(B)***SM* 593, *SM* 593-Δ*dltD*, *SM* 593Δ*dltD*-c RT-qPCR for *dlt* operon; **(C)***SM*UA159, *SM*UA159-Δ*dltD*, *SM*UA159-Δ*dltD*-c protein content; **(D)***SM*UA159, *SM*UA159Δ*dltD*, *SM*UA159Δ*dltD*-c RT-qPCR for *dlt* operon. (n=3, Data are presented as the mean ± SD, *P<0.05, **P <0.01, same lowercase letters in the same bar chart indicate no statistical difference, and different lowercase letters indicate statistical difference, P<0.05).

The results of the BCA protein assay revealed a significant decrease in protein content in the *SM* 593-Δ*dltD* biofilm (P<0.01; **Figure 7A**), whereas *SM*UA159-Δ*dltD* exhibited a significant increase (P<0.01; **Figure 7C**). RT–qPCR analysis of *dlt* operon expression revealed significant downregulation in *SM* 593-Δ*dltD* (P<0.01; **Figure 7B**) and upregulation in *SM*UA159-Δ*dltD* compared with the wild-type strains (P<0.01; **Figure 7D**).

### 3.3 *dltD* deletion impairs acid production and tolerance in *Streptococcus mutans* biofilms

**Figure 8:**
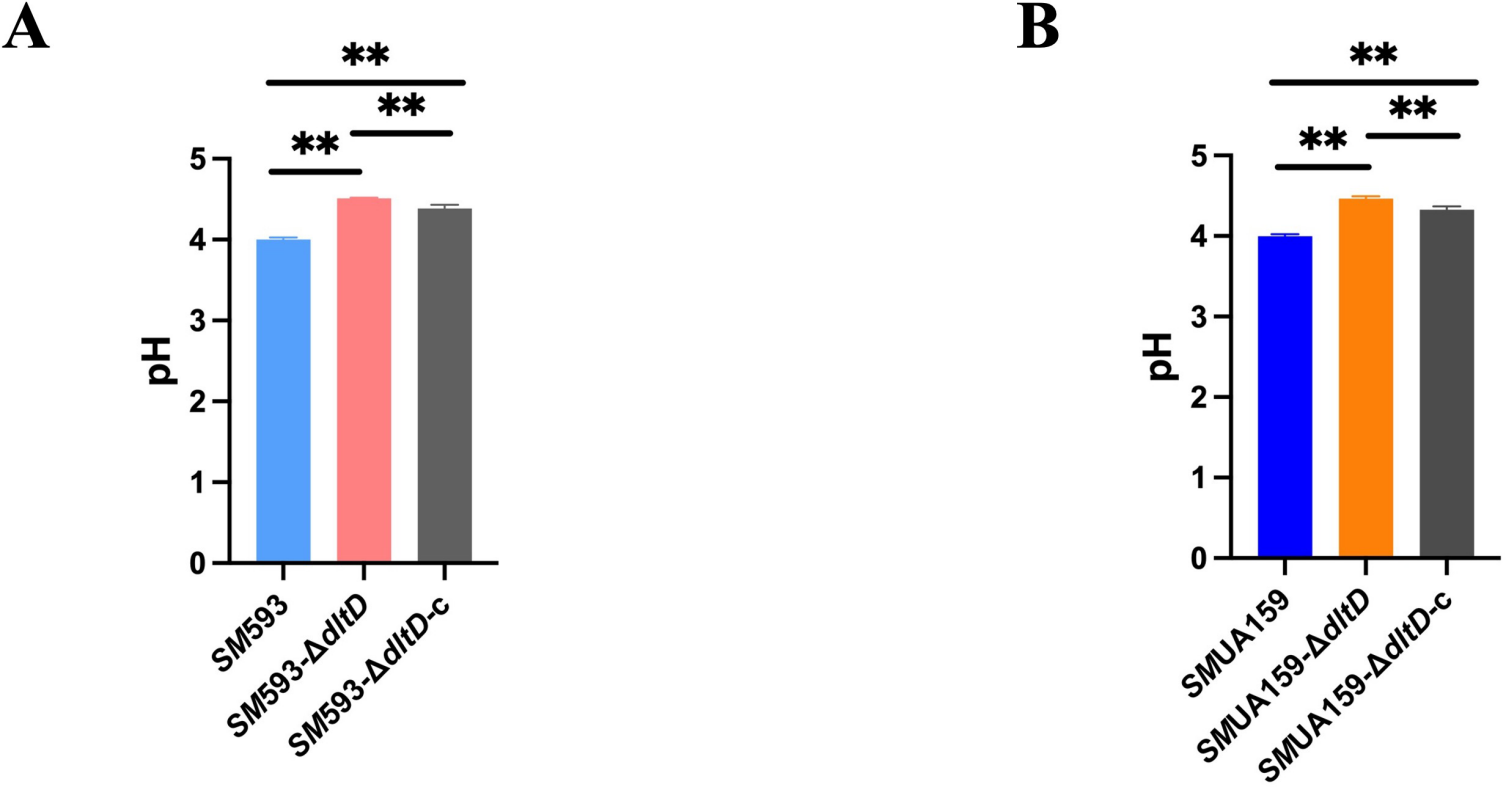
**(A)** SM 593, SM 593-DdltD, SM 593-DdltD-c acid production; **(B)** SMUA159, SMUA159-DdltD, SMUA159-DdltD-c acid production. (n=3, Data are presented as the mean ± SD, **P <0.01).

**Figure 9:**
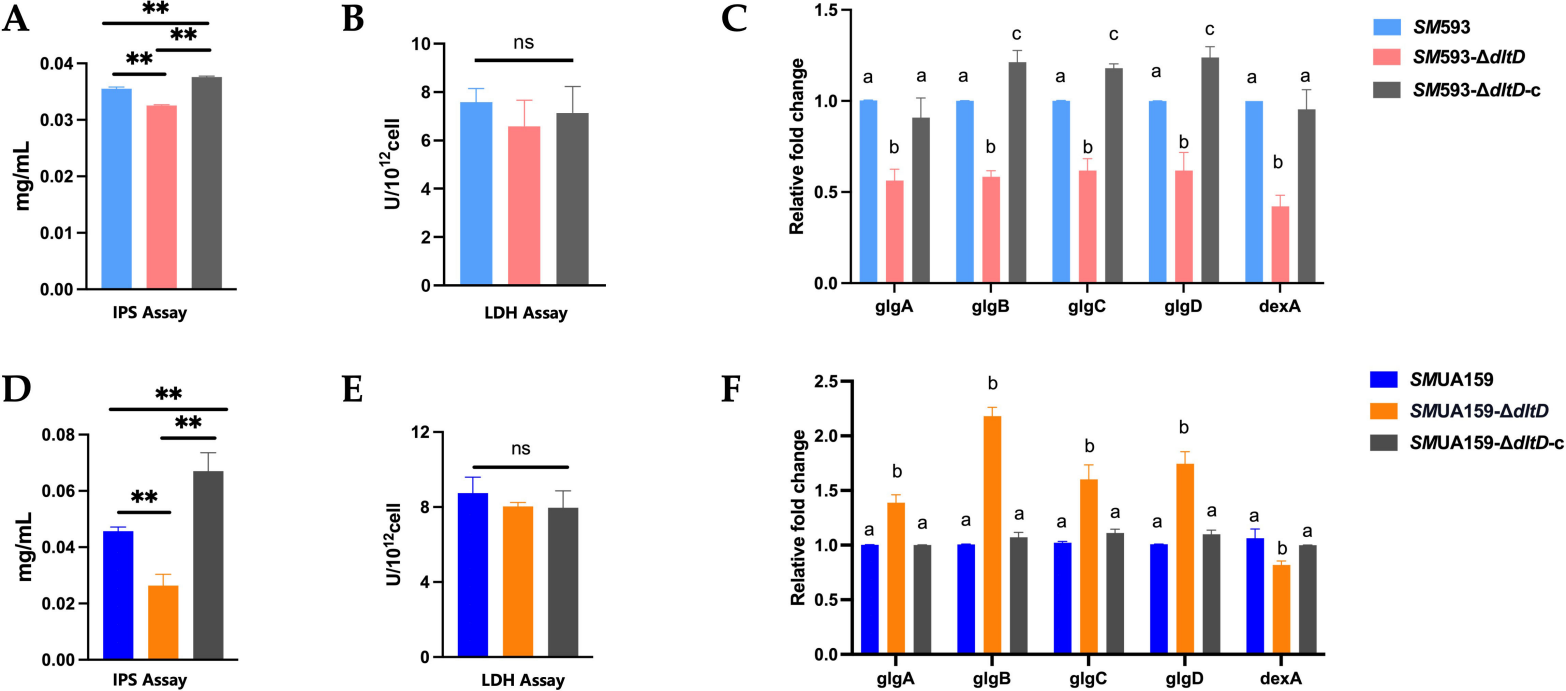
**(A)***SM* 593, *SM* 593-Δ*dltD*, *SM* 593-Δ*dltD*-c IPS content; **(B)***SM* 593, *SM* 593-Δ*dltD*, *SM* 593-Δ*dltD*-c LDH activity; **(C)***SM* 593, *SM* 593-Δ*dltD*, *SM* 593-Δ*dltD*-c RT-qPCR for IPS and carbohydrate metabolism; **(D)***SM*UA159, *SM*UA159-Δ*dltD*, *SM*UA159-Δ*dltD*-c IPS content; **(E)***SM*UA159, *SM*UA159-Δ*dltD*, *SM*UA159-Δ*dltD*-c LDH activity; **(F)***SM*UA159, *SM*UA159-Δ*dltD*, *SM*UA159-Δ*dltD*-c RT-qPCR for IPS and carbohydrate metabolism. (n=3, Data are presented as the mean ± SD, **P <0.01, same lowercase letters in the same bar chart indicate no statistical difference, and different lowercase letters indicate statistical difference).

A biofilm acid production assay revealed a significant reduction in the acidogenicity of the *dltD* mutant compared with those of the wild-type strains (P<0.01; **Figures 8A, B**). IPS quantification revealed a marked decrease in IPS content in the *dltD* mutant (P<0.05; **Figures 9A, D**). Lactate dehydrogenase (LDH) activity assays revealed no statistically significant difference between the *dltD* mutant and the wild-type strains (**Figures 9B, E**). RT–qPCR analysis revealed downregulation of the expression of *dexA*, encoding a dextranase responsible for glucan degradation (Florez Salamanca and Klein, 2018), in the *dltD* mutant (P<0.05). The expression of the *glg* operon, which mediates IPS synthesis (Costa Oliveira et al., 2021), was significantly decreased in *SM* 593-Δ*dltD* (P<0.05; **Figure 9C**) but increased in *SM*UA159-Δ*dltD* (P<0.05; **Figure 9F**).

**Figure 10:**
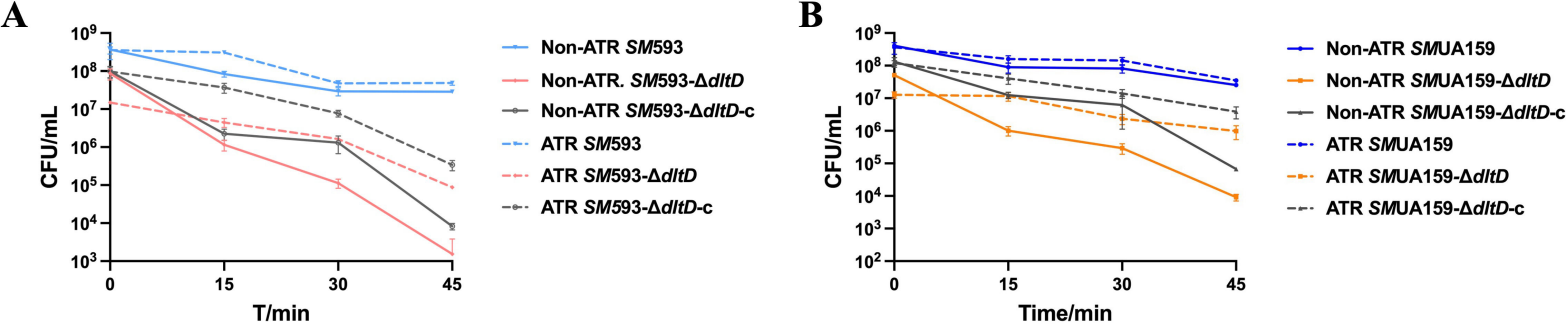
**(A)** SM 593, SM 593-DdltD, SM 593-DdltD-c time-survival curve of biofilm in pH 3.5 conditions; **(B)** SMUA159, SMUA159-DdltD, SMUA159-DdltD-c time-survival curve of biofilm in pH 3.5 conditions.

An acid tolerance assay demonstrated that compared with the wild-type strains, the *dltD* mutant exhibited reduced acid resistance (**Figures 10A, B**). However, after preacidification at pH 5.5, the *dltD* mutant retained acid tolerance response (ATR) capability (**Figures 10A, B**).

**Figure 11:**
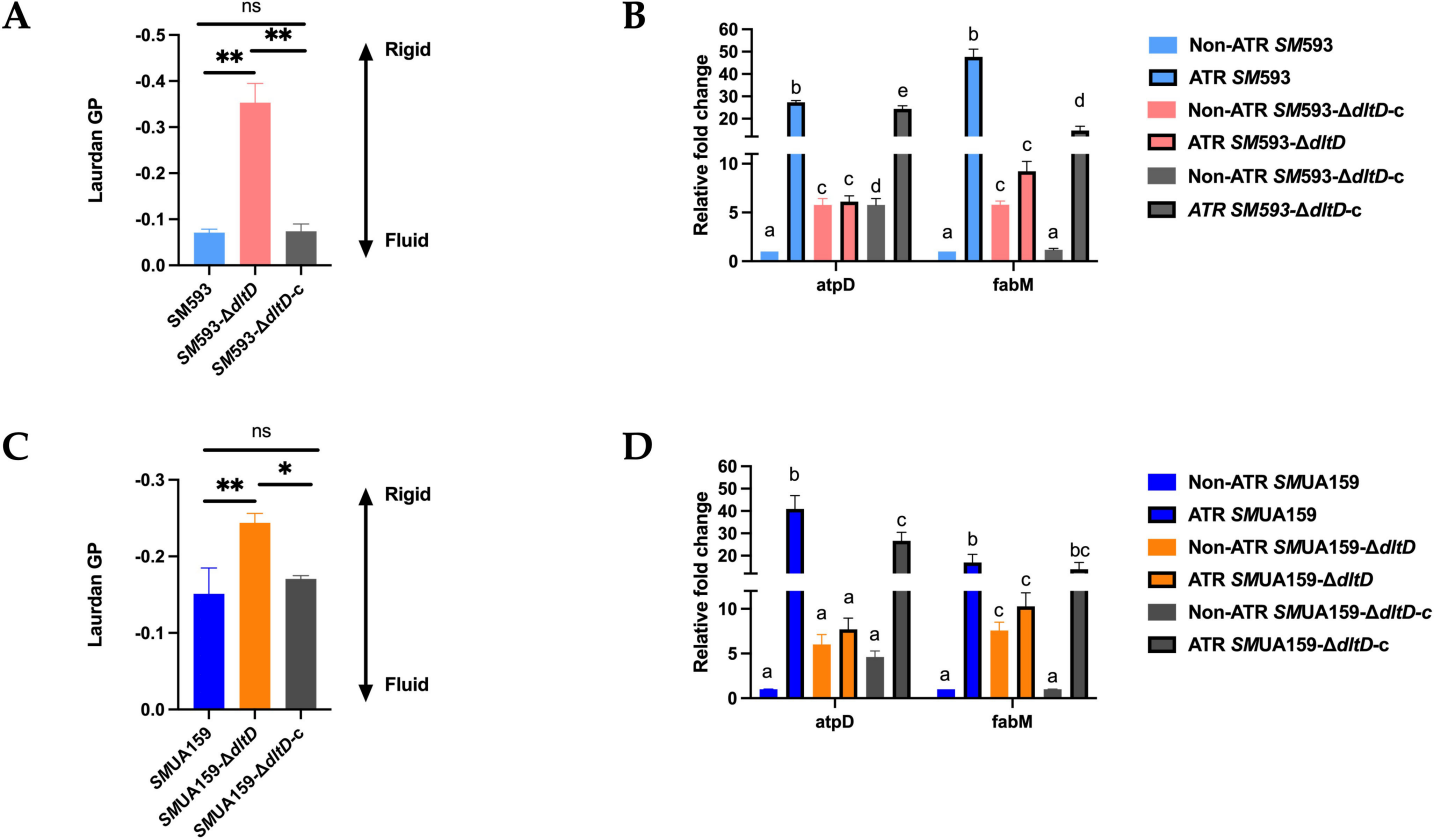
**(A)***SM* 593, *SM* 593-Δ*dltD*, *SM* 593-Δ*dltD*-c cell membrane fluidity in pH 5.5 condition; **(B)***SM* 593, *SM* 593-Δ*dltD*, *SM* 593-Δ*dltD*-c RT-qPCR for acid tolerance related genes under neutral and pH5.5 condition; **(C)***SM*UA159, *SM*UA159-Δ*dltD*, *SM*UA159-Δ*dltD*-c cell membrane fluidity in pH 5.5 condition; **(D)***SM*UA159, *SM*UA159-Δ*dltD*, *SM*UA159-Δ*dltD*-c RT-qPCR for acid tolerance related genes under neutral and pH 5.5 conditions. (n=3, Data are presented as the mean ± SD, *P <0.05, **P <0.01, same lowercase letters in the same bar chart indicate no statistical difference, and different lowercase letters indicate statistical difference).

Under acidic conditions (pH 5.5), compared with the wild-type strains, the *dltD* deletion strain displayed significantly reduced membrane fluidity (P<0.01; **Figures 11A, C**). RT–qPCR was performed to analyze the expression of *atpD*, encoding the β-subunit of H^+^-ATPase, and *fabM*, which is involved in unsaturated fatty acid synthesis (Chamlagain et al., 2024), under neutral (pH 7.0) and acidic (pH 5.5) conditions. In the wild-type strains, acidic conditions induced significant upregulation of both *fabM* and *atpD* (P<0.05; **Figures 11B, D**). Although the *dltD* mutant tended toward increased *fabM* and *atpD* expression under acid stress, these changes were not statistically significant. Notably, under acidic conditions, compared with the *dltD* deletion strain, the wild-type strains presented significantly higher *fabM* and *atpD* expression levels than the *dltD* deletion strain (P<0.05; **Figures 11B, D**).

## 4 Discussion

The deletion of the *dltD* gene results in attenuated cariogenicity of *SM* isolates from various sources. This may be ascribed to alterations in the composition of the biofilm extracellular matrix and modifications in metabolic activity. However, discrepancies in regulation mode exist among different strains.

In *SM*UA159-Δ*dltD*, the reduced caries severity in the rat model aligns with the observations of a study by Castillo et al.’s (Castillo Pedraza et al., 2019) on diminished virulence. Similarly, *SM* 593-Δ*dltD* exhibited decreased cariogenicity, particularly in sulcal and proximal caries, mirroring its impaired biofilm formation capabilities. These findings collectively implicate *dltD* as a critical regulator of *SM* pathogenicity, although its mechanistic role differs across strains. In the *SM*UA159-derived strains, *SM* colonization on tooth surfaces was greater in *SM*UA159 than in *SM*UA159-Δ*dltD*, which is consistent with the caries scores and *in vitro* adhesion results. Interestingly, in the *SM* 593-derived strains, the total number of plaque bacteria was significantly lower than that in controls. No significant differences in *SM* colonization amount or proportion were observed between *SM* 593 and *SM* 593-Δ*dltD*.

The ecological plaque hypothesis posits that dental caries arises from dynamic stability to metabolic imbalance, leading to an increased proportion of acid-producing and acid-tolerant bacterial communities within the plaque (Zhang et al., 2025). *SM* 593, recovered from enamel adjacent to pit-and-fissure caries in a patient with high caries susceptibility but optimal oral hygiene, exhibited robust *in vitro* biofilm formation, acidogenesis, and acid tolerance—hallmarks of pathogenicity in cariogenic streptococci (Jiang et al., 2017). These traits suggest that *SM* 593 may disrupt plaque homeostasis by outcompeting commensal species, potentially driving microbial dysbiosis. Although speculative, this hypothesis aligns with evidence that acidogenic/aciduric taxa can displace less virulent bacteria through competitive exclusion, particularly in carbohydrate-rich niches (Fakhruddin et al., 2022). However, direct validation requires coculture competition assays to quantify the impact of *SM* 593 on plaque community structure—a critical gap in the current findings. Recent high-throughput sequencing revealed that ≥70% of oral bacteria are culturable (Balachandran et al., 2020), and unculturable taxa may still influence the ecological success of *SM* 593. The bacterial quantification method employed in this study was CFU, with statistical results reflecting only culturable species in dental plaque, which presents certain limitations in bacterial load statistics. Future studies integrating metagenomic and metabolomic profiling will clarify whether *SM* 593-mediated dysbiosis stems from direct inhibition or metabolic synergy with cocolonizers.

Compared with wild-type *SM* 593, *SM* 593-Δ*dltD* exhibited enhanced adhesion capabilities compared to the wild-type *SM* 593. This phenotypic advantage may facilitate early colonization of tooth surfaces by outcompeting commensal streptococci under carbohydrate-rich conditions (Wei et al., 2024). However, the mutant strain demonstrated a reduction in WIP content in the biofilm, a critical structural component for maintaining biofilm cohesion and acid resistance, was reduced in the mutant strain. Such depletion likely compromises structural integrity during late-stage biofilm development, potentially explaining the observed decrease in viable biomass under prolonged acidic challenge. Notably, *SM* 593-Δ*dltD* exhibited elevated autolysis rates (Du et al., 2024), which while providing nutrient cross-feeding opportunities for cohabiting taxa, simultaneously accelerated self-eradication through lysis (Dong et al., 2024). The interplay of these phenotypic variations—enhanced early colonization offset by compromised structural persistence and accelerated population decline—creates a homeostatic equilibrium that negates significant differences in *SM* colonization density between strains. Future studies could focus on elucidating the precise mechanisms underlying these complex interactions, perhaps through real-time imaging of biofilm formation and more detailed biochemical analysis of the biofilm matrix components at different stages of development.

Conversely, in a rat caries model, the *SM* 593-Δ*dltD* showed a downward trend in buccal caries scores but demonstrated a significantly decreased in sulcal and proximal caries scores. The smooth surfaces of teeth exhibit stronger self-cleaning effects. Compared with rougher dental surfaces such as sulcal or proximal areas, such anatomical characteristics reduce plaque retention and caries susceptibility. Although the adhesion ratio of *SM 593-ΔdltD* increased, its acid production capacity significantly decreased. These factors collectively contributed to the lack of significance in the buccal caries score. The anatomical characteristics of the sulcal and proximal surfaces are conducive to bacterial colonization. Once established in these niches, bacteria can drive accelerated caries progression through localized acid production and biofilm maturation. In our rat model, buccal caries mainly involved mainly enamel and shallow dentin, where environmental buffering and clearance mechanisms may mitigate acid-mediated damage. The sulcal and the proximal surfaces facilitate bacterial colonization, thereby promoting caries progression. As caries advances, the progressive acidification of the microenvironment (i.e., pH decline) enhances the pathogenic potential of *SM* (Spatafora et al., 2024), which in turn exacerbates lesion development.

Compared with the wild-type strains, the *dltD* mutant exhibited marked reductions in viable cell viability and metabolic activity within biofilms, suggesting impaired ecological fitness. Quantitative analysis revealed a significant reduction in WIP in the *dltD* mutant biofilm, a critical determinant for biofilm cohesion and acid resistance. Notably, *SM*UA159-Δ*dltD* demonstrated an increased WSP, whereas *SM* 593-Δ*dltD* showed no significant WSP alteration. This strain-specific EPS metabolism exhibits functional divergence analogous that of YidC paralogs—the conserved YidC/Oxa1/Alb3 family of membrane-integrated chaperones and insertases—where *yidC1* deletion reduces WIP without altering WSP, and *yidC2* deletion increases WSP despite WIP loss (Mishra and Brady, 2021). These observations suggest that although the *dltD* gene is conserved across *SM* strains, it may exert strain-specific regulatory effects on EPS metabolism—analogous to the functional divergence observed between the *yidC1* and *yidC2* paralogs.

In *SM*, sucrose metabolism via glucosyltransferases (Gtfs) drives biofilm formation through dual polysaccharide synthesis: WIP, mediates adhesion and structural integrity, whereas WSP, serves as an energy reservoir for acid persistence (Ren et al., 2022). The *gtfB* (WIP synthesis) and *gtfC* (dual WIP/WSP synthesis) genes, which are cotranscribed under shared regulatory control, are critical for microcolony formation and interbacterial aggregation (Zhang et al., 2021). In contrast, *gtfD* (WSP synthesis) operates independently upstream of this operon, enabling strain-specific metabolic flexibility (Zhang et al., 2021). In our study, RT–qPCR revealed that, compared with the wild-type strains, the *dltD* mutant significantly downregulated the expression of *gtfB* and *gtfC*, while *gtfD* expression remained unchanged. These findings support the hypothesis that *dltD* deletion in *SM* inhibits *gtfB* and *gtfC* transcription, which in turn leads to a significant reduction in biofilm WIP content and subsequent alterations to biofilm structure. However, the *SM*UA159-Δ*dltD* mutant maintained the *gtfD* expression level, potentially compensating for WIP loss through alternative metabolic pathways. In *SM*, *gbpA* and *gbpB* encode glucan-binding proteins (Chen et al., 2022). GbpA binds to EPS, participates in the formation of 3D biofilm structures, provides a favorable microenvironment for biofilm-resident bacteria (**Supplementary File**), and promotes bacterial resistance to external stress. GbpB mainly maintains cell wall construction and surface hydrophobicity (Cugini et al., 2019). Studies have also shown that GbpA is involved in cell wall construction and cell shape maintenance. The *gbpA*/*gbpB* mutant forms loose, structurally uneven biofilms (Cugini et al., 2019). RT–qPCR revealed, compared with the wild-type strains, the *dltD* mutant strains presented significantly downregulated expression levels of *gbpA* and *gbpB*. This may cause the *dltD* mutant to be less prone to colonizing the buccal side for biofilm formation. Taking the above factors into account, this may explain why the *dltD* mutant only trended toward reduced buccal caries but caused significantly fewer sulcal and proximal caries.

In biofilm matrices, eDNA mainly stems from vesicle release and bacterial autolysis. Autolysis, defined as the breakdown of microorganisms by endogenous hydrolases such as autolysins, leads to the release of intracellular components (Park et al., 2022; Rohde, 2019). In the gram-positive bacteria *Bacillus licheniformis*, the deletion of *dltD* increases the negative charge on the bacterial surface, leading to elevated autolysis levels and a reduction in viable cell counts within biofilms (Mo et al., 2019). Conversely, in the *Staphylococcus epidermidis atlE* mutant, decreased autolysis corresponds to reduced eDNA release and diminished biofilm formation (Wang et al., 2023). Our findings revealed that compared with the wild-type strains, the *dltD* mutant exhibited enhanced autolysis, a significant increase in eDNA content within biofilms, and a notable upregulation in the expression of the autolysin-related gene *atlE*. These results suggest that the substantial decrease in viable cell numbers and the decline in metabolic activity within biofilms are associated with the elevated bacterial autolysis in the *dltD* mutant. These findings establish a novel link between *dltD*-mediated LTA modification and autolysis-driven biofilm dynamics, advancing our understanding of *SM* pathogenesis in cariogenic biofilms.

Comparative analysis of extracellular matrix (ECM) protein dynamics in *SM* biofilms revealed strain-specific regulatory patterns: compared with the wild-type strain, *SM*UA159-Δ*dltD* exhibited ECM protein enrichment, whereas *SM* 593-Δ*dltD* showed a reduction in ECM protein enrichment. This divergence is correlated with the differential expression of the *dlt* operon—upregulated expression level in *SM*UA159-Δ*dltD* but downregulated expression level in *SM* 593-Δ*dltD*. In *Bacillus licheniformis*, the deletion of *dltD* increased extracellular protein secretion (He et al., 2019). In *Bacillus* species, upregulated expression of the *dlt* operon enhances extracellular protein secretion (Mo et al., 2019). Midian C et al. demonstrated that the ECM of biofilms formed by the *SM*UA159-Δ*dltD* mutant strain exhibits increased extracellular LTA and protein content (Castillo Pedraza et al., 2017). The authors suggested that deletion of *dltD* within the *dlt* operon may activate compensatory pathways, leading to enhanced extracellular release of LTA and proteins by these strains (Castillo Pedraza et al., 2017). Therefore, we hypothesized that the expression level of the *dlt* operon is correlated with the extracellular protein level in *SM*. However, the molecular mechanisms underlying the different regulatory patterns of the *dlt* operon in these two strains need further study.

Acidogenic and acid-tolerant capabilities represent major cariogenic virulence factors of *SM*. Despite its low abundance within carious dental plaque, *SM* demonstrates significant ecological expansion as caries advance, inversely correlating with the depletion of acid-sensitive bacterial populations (Zhang et al., 2022). The acidogenic potential of this species stems from carbohydrate metabolism, predominantly the generation of lactic acid via glycolytic pathways (Lemos et al., 2019). Carbohydrate acquisition through phosphotransferase (PTS) and non-PTS systems fuels glycolysis, yielding adenosine triphosphate (ATP) and essential biomolecules while maintaining intracellular pH homeostasis (Lv et al., 2025). The synthesis of EPS and IPS further optimizes carbon utilization under nutrient-limited conditions. IPS, primarily serves as a critical glycolytic substrate during carbohydrate scarcity (Costa Oliveira et al., 2021). Sustained acidification (pH <5.5) induces enamel demineralization through calcium phosphate dissolution, initiating carious lesion development (Lemos et al., 2019). Comparative analysis of acidogenicity in *SM* strains revealed distinct metabolic adaptations in the *dltD* mutants. Compared with the wild-type strain, *SM*UA159-Δ*dltD* and *SM* 593-Δ*dltD* exhibited a reduction in acid production capacity. This metabolic divergence correlated with the differential regulation of carbohydrate metabolism pathways. RT–qPCR demonstrated downregulation of the expression of *dexA* (encoding a glucan-degrading enzyme) in the *dltD* mutants, impairing the intracellular oligosaccharide uptake required for IPS synthesis via the *glg* operon. Notably, despite reduced IPS levels, *SM*UA159-Δ*dltD* exhibited upregulated expression of the *glg* operon (encoding IPS synthesis enzymes), whereas *SM* 593-Δ*dltD* showed downregulated *glg* operon expression, mirroring the IPS content reduction. The *dlt* operon of *SM*UA159, located upstream of the *glg* operon, can positively regulate the expression level of the *glg* operon (Spatafora et al., 1999). The results of this study further confirmed this conclusion, as the *dlt* operon could also positively regulate the expression of the *glg* operon in two different *SM* strains. Therefore, it is hypothesized that in *SM* 593-Δ*dltD*, the downregulated expression of the *dexA* gene reduces the oligosaccharides involved in intracellular metabolism. Concurrently, the downregulated expression of the *glg* operon leads to decreased IPS synthesis capacity, reducing the metabolic substrates available for bacterial glycolysis, which is one of the reasons for the reduced acid-producing capacity. Nevertheless, in *SM*UA159-Δ*dltD*, although the IPS content decreases, the expression level of the *glg* operon is upregulated. We speculate that this might be a compensatory response following the reduction in IPS synthesis in this strain; however, the specific reason requires further investigation. These findings establish a dual regulatory axis in which *dltD* deletion differentially influences carbon metabolism through strain-specific interactions between LTA modification systems and carbohydrate utilization pathways.

At a pH of 5.5, *SM* activates adaptive acid tolerance mechanisms, including membrane proton barrier reinforcement and stress protein synthesis. Key acid tolerance response (ATR) components include (1) reduced proton permeability via lipid bilayer modifications, (2) enhanced ATP-driven proton extrusion, (3) membrane fluidity optimization through fatty acid remodeling, and (4) molecular chaperone production to stabilize macromolecular structures (Boisen et al., 2021; Li et al., 2022). H^+^-ATPases are membrane-bound proton transporters regulated by the *atp* operon. The upregulated expression of the *atp* operon in acidic environments enables the extrusion of excess cytoplasmic H^+^, a crucial process for maintaining intracellular pH homeostasis (Li et al., 2022). Increasing membrane fluidity and preserving intracellular pH homeostasis are key adaptive mechanisms that *SM* employs to survive in acidic environments. These adaptations enable *SM* to thrive in cariogenic microenvironments, driving localized decreases in pH that are critical for progressive caries pathogenesis. Deletion of the *dltD* impaired the acid tolerance of the *SM* biofilms. However, the biofilm ATR capability follows preacidification. Our findings demonstrated that, compared with the wild-type strain, the *dltD* mutant strain exhibited significantly impaired responsiveness of both the *atpD* (H^+^-ATPase subunit) and *fabM* (unsaturated fatty acid synthase) genes under acidic conditions (pH = 5.5). Concurrently, the mutant displayed a reduction in membrane fluidity, which was attributed primarily to decreased unsaturated fatty acid composition. Collectively, these findings suggest that the deletion of *dltD* leads to the downregulated expression of the UFA synthesis-related gene *fabM*, which reduces membrane fluidity, and the proton efflux-related gene *atpD*, which impairs the proton pump function. These combined effects contribute to the decreased acid tolerance of the *dltD* mutant.

Taken together, our findings demonstrate that the deletion of the *dltD* in *SM* significantly attenuates its cariogenic potential. This attenuation is manifested by a marked increase in autolysis levels, a reduction in viable cell counts and metabolic activity within biofilms, and decreases in both the acid-producing and acid-tolerant capabilities of the biofilms. These changes likely disrupt optimal biofilm development and attachment, as evidenced by the varying degrees of reduced virulence observed in a rat caries model.

**Figure 12:**
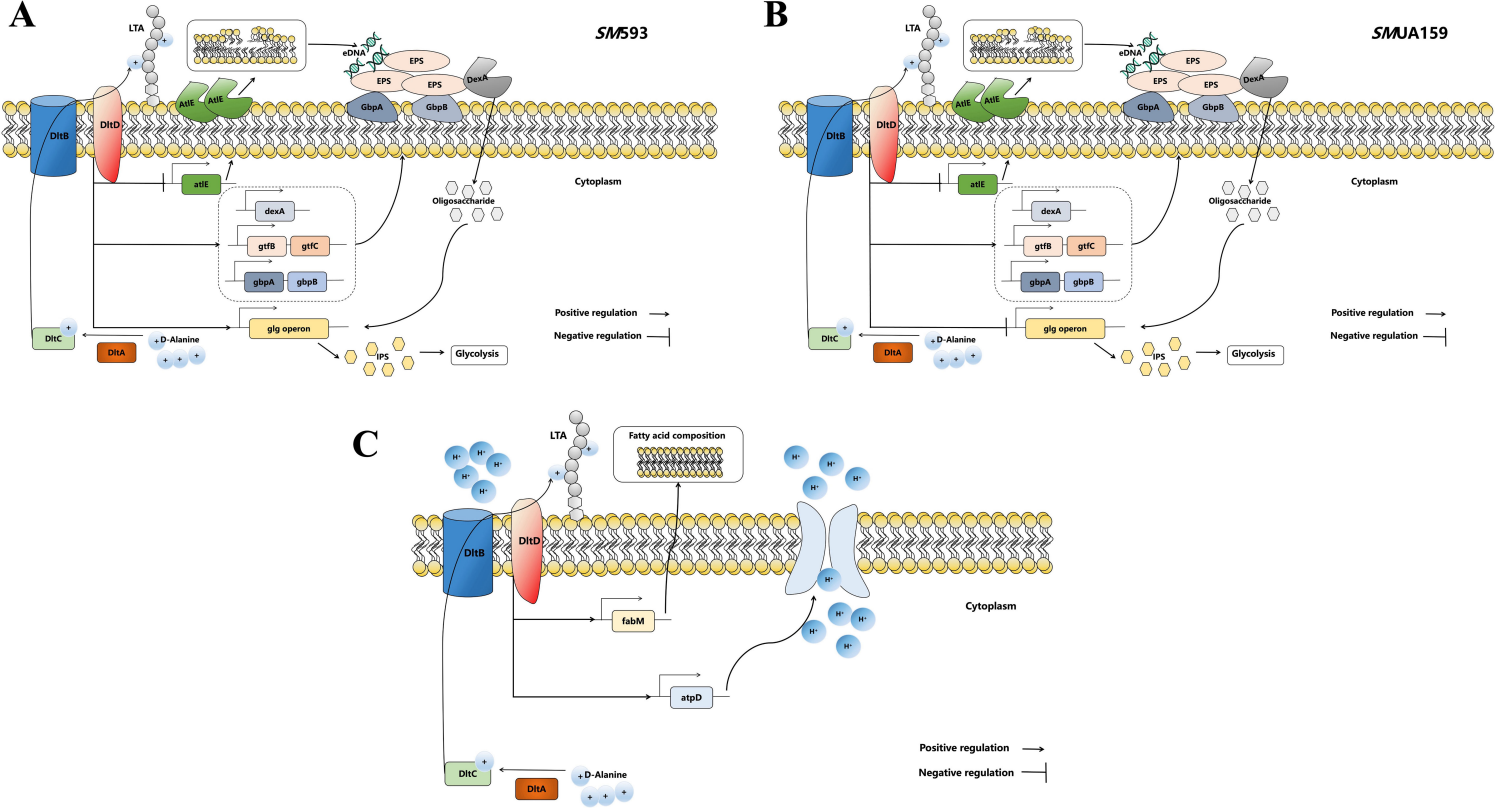
**(A)** The regulatory mode of the *dltD* gene on biofilm and IPS in *SM* 593; **(B)** The regulatory mode of the *dltD* gene on biofilm and IPS in *SM*UA159; **(C)** The regulatory mode of the *dltD* gene in the acid tolerance process of *SM*.

Notably, differences in gene regulatory mode and alterations in biofilm components exist among distinct *SM* strains (**Figure 12**). Despite these strain-specific variations, our study clearly revealed that *dltD* deletion does not impede the growth of *SM*. Instead, it specifically and substantially impairs the cariogenicity of the bacterium. This research provides a robust theoretical basis for considering the *dltD* as a promising target for ecological caries prevention strategies. By selectively targeting the *dltD* gene, it may be possible to reduce the cariogenic potential of *SM* without disrupting the overall oral microbiota balance, thus offering a novel approach to caries prevention.

## Data Availability

The original contributions presented in the study are included in the article/**Supplementary Material**. Further inquiries can be directed to the corresponding authors.
