# Supplementary material for: Deletion of dltD gene modulates biofilm matrix and acid metabolism to attenuate Streptococcus mutans cariogenicity
Source: Front Cell Infect Microbiol. 2026 Feb 16;15:1741359. doi: 10.3389/fcimb.2025.1741359 (PMC12950589; doi:10.3389/fcimb.2025.1741359)
Supplement: Supplementary file 1 [file DataSheet1.pdf]

Materials and methods:

1. Biofilm CFU

After biofilm formation, specimens were rinsed three times with PBS. The biofilm was resuspended in an equal volume of PBS. The pellet obtained after centrifugation of the bacterial suspension was rinsed three times with phosphate-buffered saline (PBS). An equal volume of PBS was then added to resuspend the bacterial cells. They were diluted 101–1010 times, and each concentration was coated on BHI agar plate with 100 μL of bacterial solution. Cells were counted after 24 h of culture under anaerobic conditions.

2. 3-(4,5-Dimethylthiazol-2-yl)-2,5-Diphenyltetrazolium Bromide (MTT) assay

After biofilm formation, specimens were rinsed three times with PBS. An equal volume of 5 mg/mL MTT solution (in PBS) was then added under light-shielded conditions, and samples were incubated at 37 °C for 4 h to allow metabolically active cells to form intracellular formazan crystals. Post-incubation, the supernatant was gently aspirated. An equivalent volume of dimethyl sulfoxide (DMSO) was added to dissolve insoluble formazan precipitates, followed by 15 min of light-protected decolorization on an orbital shaker (150 rpm). Finally, 200 μL aliquots of the solubilized solution were transferred to a 96-well microplate. OD_{570nm} was performed using a microplate reader to quantify biofilm metabolic activity.

3. Analysis of biofilm by confocal laser scanning microscopy (CLSM)

After experimental strains formed biofilms on circular glass coverslips, specimens were aseptically rinsed three times with PBS to remove planktonic cells. They were then fixed in 2.5% glutaraldehyde solution (light-protected) at 4°C for 2 h. For bacterial viability assessment, fixed biofilms were incubated with the LIVE/DEAD™ BacLight™ Bacterial Viability Kit (Thermo Fisher) for 15 min under light protection—this allowed simultaneous visualization of viable (green fluorescence) and dead (red fluorescence) cells. Processed specimens were mounted on glass slides using anti-fade medium and analyzed via CLSM (Leica TCS SP8). Three random fields per sample were imaged with a 63× oil-immersion objective (z-stack interval: 0.5 μm). 3D reconstruction and biofilm thickness analysis were performed using ImageJ. Fluorescence intensity was quantified via ImageJ's threshold-based particle analysis,

with values normalized to background signals.

4. eDNA assay

The biofilms were rinsed with PBS, followed by the addition of 1 mL PBS to each well for biofilm collection. The harvested biofilm suspension was subjected to centrifugation at 4000 rpm for a duration of 20 minutes. Subsequently, the supernatant was filtered using a filter membrane with a pore size of 0.22 μm. A 50 μL aliquot of the supernatant was mixed with an equal volume of SYTOX Green, and the mixture was incubated in the dark for 30 minutes. Upon completion of incubation, a microplate reader was employed to determine the fluorescence intensity at an excitation wavelength (λex) of 488 nm and an emission wavelength (λem) of 538 nm.

Table 1. Nucleotide sequences of primers were used in this study.

Primer	Sequence (5'-3')	Description	Reference
atlE-F	5'-AGCTGGTCCCAAAGGAAATC-3'	Autolysin	(Khan et al., 2017)
atlE-R	5'-GCCTGTGCCCAATAATCATC-3'		

Results:

1. *dltD* Gene Deletion Impaired Biofilm Formation in *Streptococcus mutans*

CFU counting revealed a significant reduction in viable bacterial numbers within the dltD mutant biofilm compared to the wild-type strains ($P<0.05$; Figs. 1A, 1C). The MTT assay further demonstrated a significant decline in biofilm metabolic activity of the dltD mutant compared to the wild-type strains ($P<0.01$; Figs. 1B, 1D). CLSM analysis showed that the green fluorescence intensity (representing viable bacteria) was markedly decreased, while red fluorescence intensity (indicating dead bacteria) was significantly increased in the *dltD* mutant biofilm relative to the wild-type strains (Fig. 2).

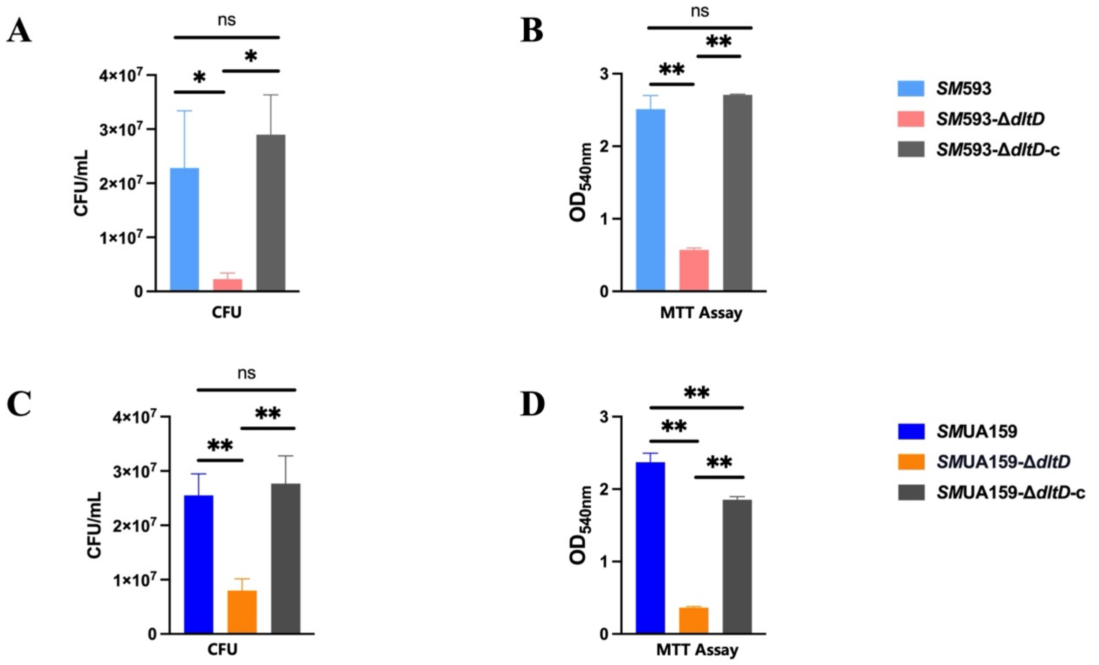

Figure 1. A: *SM*593, *SM*593Δ*dltD*, *SM*593Δ*dltD*-c CFU counting; B: *SM*593, *SM*593Δ*dltD*, *SM*593Δ*dltD*-c metabolic activity; C: *SM*UA159, *SM*UA159Δ*dltD*, *SM*UA159Δ*dltD*-c CFU counting; D: *SM*UA159, *SM*UA159Δ*dltD*, *SM*UA159Δ*dltD*-c metabolic activity. (n=3, Data are presented as the mean±SD, *P<0.05, ** P <0.01).

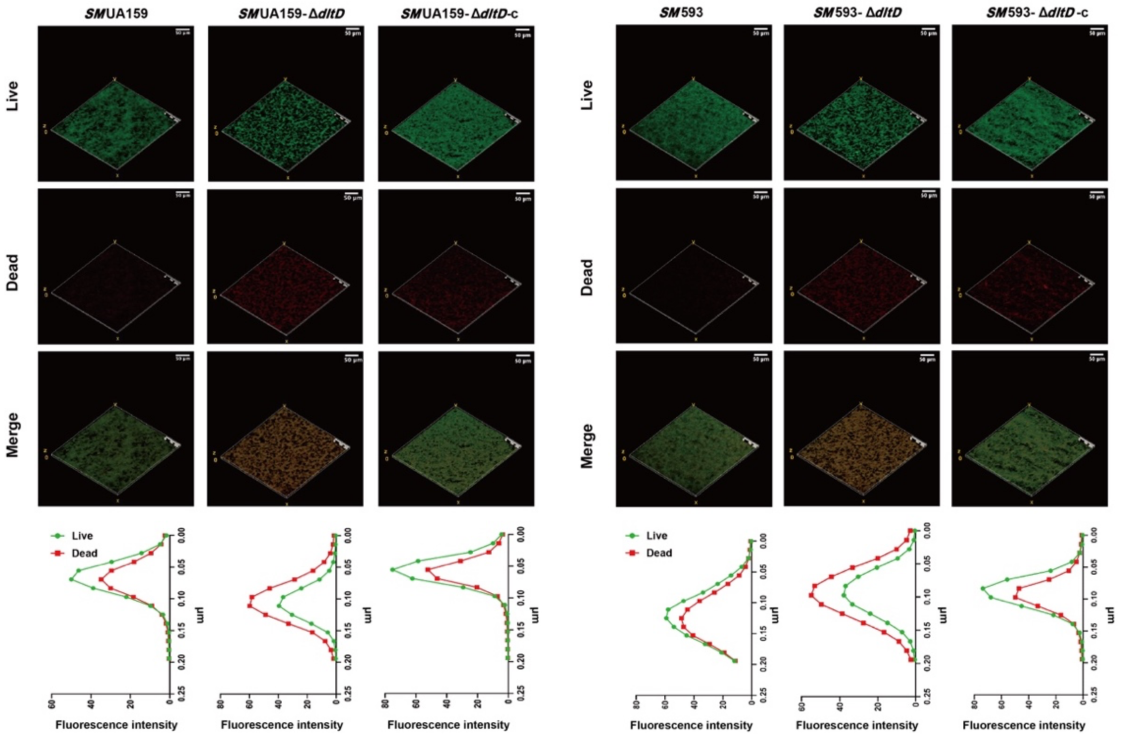

Figure 2. CLSM image of biofilm: green fluorescence indicates living bacteria, and red fluorescence indicates dead bacteria.

2. *SM*UA159-Δ*dltD* biofilm eDNA level increased

Extracellular DNA (eDNA) quantification revealed a significant increase in eDNA content in the *SM*UA159-Δ*dltD* biofilm compared to the wild-type strains (P<0.01; Fig. 3A). Consistent with this, RT-qPCR showed significantly upregulated expression of the autolysis-associated gene *atlE* in *SM*UA159-ΔdltD (P<0.01; Fig. 3B), mirroring results observed in *SM*593-Δ*dltD*.

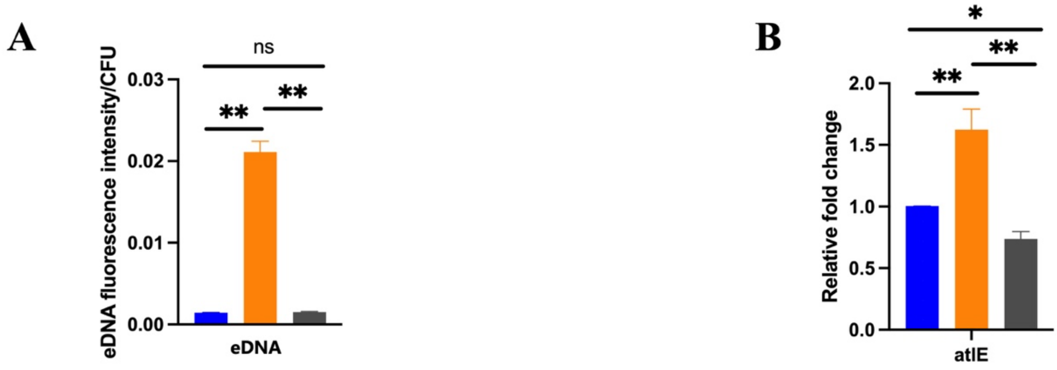

Figure 3. A: eDNA content; B: RT-qPCR for *atlE*. (n=3, Data are presented as the mean±SD, *P<0.05, ** P <0.01).
